# A cartridge-based assay for improved detection of multidrug-resistant *Mycobacterium tuberculosis* directly from sputum

**DOI:** 10.1128/jcm.01100-25

**Published:** 2026-03-30

**Authors:** Paulami Rudra, Heta Parmar, Naranjargal Daivaa, Claire Tran, Anshika Narang, Simranjit Singh, Dhara Somaiya, Samarpita Roybardhan, Ritu Anwesa, Robert Reiss, Shawn Ward, Adam Penn-Nicholson, Margaretha De Vos, Derek T. Armstrong, April L. Borkman, Jerrold Ellner, Susan E. Dorman, Soyeon Kim, Lin Yuan, David Alland, Soumitesh Chakravorty

**Affiliations:** 1 Department of Medicine, Rutgers New Jersey Medical School 214907, Newark, New Jersey, USA; 2 Cepheid Inc. 60159 , Sunnyvale, California, USA; 3 Frontier Science Foundation, Brookline, Massachusetts, USA; 4 Foundation for Innovative New Diagnostics 91635, Geneva, Switzerland; 5 Johns Hopkins School of Medicine 1500 , Baltimore, Maryland, USA; 6 Medical University of South Carolina 2345, Charleston, South Carolina, USA; Vanderbilt University Medical Center, Nashville, Tennessee, USA

**Keywords:** molecular diagnostics, multidrug resistant, tuberculosis

## Abstract

Tuberculosis (TB) is a global health threat complicated by increasing INH resistance (INH-R) and rifampicin resistance (RIF-R), including RIF-R due to *rpoB* Ile491Phe (I491F) mutations, which are not detected by current molecular resistance tests. We developed a low‐complexity automated nucleic acid amplification test MDRmDx, designed to operate on the 10-color GeneXpert instrument, which detects *Mycobacterium tuberculosis* (MTB), and RIF-R in both the rifampicin resistance determining region (RRDR) and the I491F allele, and INH-R due to mutations in *katG*, *fabG1*, and the *inhA* promoter directly from sputum. In an analytic study, the limit of detection (LoD) for detecting MTB in sputum was 21.6 CFU/mL (95% confidence interval [CI]: 14.9–28.3) for the MDRmDx assay, versus 19.1 CFU/mL (95% CI: 14.0–24.1) for the Xpert MTB/RIF Ultra assay (Ultra). The LoD for rifampicin susceptibility (RIF-S) detection was 108.8 CFU/mL (95% CI: 77.4–140.2) versus 100.9 CFU/mL (95% CI: 67.3–134.5) for MDRmDx versus Ultra. The LoD for INH susceptibility detection was 92.2 CFU/mL (95% CI: 65.3–119.1). MDRmDx identified all *rpoB* mutations with a ≥0.5% global prevalence, including several not detected by Ultra. MDRmDx was 100% specific. Testing frozen sputum enriched for smear-negative, RIF-R, and INH-R samples, MDRmDx sensitivity was 91.3% (95% CI: 85.6–94.8) for MTB detection, 100% (95% CI: 95.1–100) for RIF resistance, and 98.7% (95% CI: 93.0–99.8) for INH resistance, with a specificity of 98.0% (95% CI: 89.3–99.6) for MTB detection, 93.8% (95% CI: 83.2–97.9) for RIF resistance, performance comparable to Ultra. MDRmDx specificity for INH-R was 100% (95% CI: 90.8–100).

**IMPORTANCE:**

The Xpert MTB/RIF Ultra assay (Ultra) is a gold standard test for rapidly diagnosing tuberculosis (TB) as well as resistance to rifampicin directly from sputum samples. However, the Ultra assay does not detect certain rifampicin resistance mutations, which are observed clinically and cannot detect resistance to isoniazid, another key drug against TB. We modified the Ultra assay, creating a prototype MDRmDx assay designed for 10-color instruments that detects isoniazid resistance, which can be a precursor of potential rifampicin resistance and has improved ability to detect rifampicin resistance. In both analytic and retrospectively collected clinical sputum samples, we found that the MDRmDx assay performed at least as well as the Ultra assay with the additional advantage of detecting isoniazid resistance and *rpoB* I491F mutations with high sensitivity and specificity. The MDRmDx assay's improved performance should improve detection of drug-resistant TB and facilitate the selection of effective treatment.

## INTRODUCTION

Tuberculosis (TB) is the leading cause of death from a single infectious disease worldwide (1). TB treatment can take many months to complete (2) and can be further complicated when *Mycobacterium tuberculosis*, the causative agent of TB, becomes drug resistant (3). Multidrug-resistant (MDR) TB, defined as resistance to both isoniazid (INH-R) and rifampicin (RIF-R), is associated with increased mortality rates. MDR-TB is responsible for 3.2% of cases with new TB diagnoses and 16% among previously treated cases (1). Rapid and accurate diagnosis of TB that is INH-R, RIF-R, or MDR is urgently needed to prevent transmission of drug-resistant strains, implement appropriate treatment regimens, and limit the evolution of additional resistance.

Culture-based phenotypic drug susceptibility testing (P-DST) of *M. tuberculosis* currently takes weeks to months before results are available to the patient, and in low-income countries, P-DST is often limited to a few centralized facilities (4). Molecular drug susceptibility tests (M-DSTs) that detect the presence of *M. tuberculosis* and drug resistance can provide this information much more quickly (5). Several low-complexity M-DST approaches are commercially available, including some that can be performed at or near point of care, such as the Molbio TrueNat (6, 7), Eiken TB-LAMP (8, 9), and Xpert MTB/RIF Ultra (Ultra) (Cepheid, USA) (10) assays.

The Ultra assay was recently shown to have the same sensitivity as a single MGIT-based culture for MTB detection (11). The Ultra assay targets the multi-copy *IS6110* and *IS1081* genes to detect TB and the 81 bp rifampicin resistance determining region (RRDR) in the *rpoB* gene to detect RIF-R. RRDR mutations account for approximately 95% of all RIF-R TB (12) and RIF-R detection has been shown to be a good surrogate for MDR (13). However, Mtb strains that are INH-R but rifampicin susceptible (RIF-S) are also associated with poor TB treatment outcomes (14). Equally concerning are recent reports highlighting the emergence of mutations outside of the RRDR (15), particularly involving *rpoB* I491F mutations, which are associated with elevated RIF minimal inhibitory concentrations (MICs) and treatment failure (16). The 2023 World Health Organization (WHO) mutation catalog suggests that I491F mutations account for approximately 0.7% of global RIF-R, and this mutation is highly specific for rifampicin resistance (17). Furthermore, a national drug resistance survey conducted in Eswatini detected a substantial increase in prevalence of I491F mutations among people with MDR-TB from 30% in 2009 to 56% in 2017–2018 driving a local MDR-TB epidemic (18–20). A study in Mozambique recently highlighted the importance of developing new tests to prevent the spread of these diagnostic escape strains (21, 22). However, a thin-layer agar (TLA) method has been the only relatively low-complexity test reported to detect *rpoB* I491F mutation with sufficient sensitivity, and this test is limited by its requirements for standard inoculum amounts, fresh sputum, and multiple time-consuming readings (23).

A survey of drug-susceptible and drug-resistant *M. tuberculosis* genomes showed that INH-R mutations preceded mutations that confer RIF-R in 96% of people with MDR-TB. This phenomenon was noted across all lineages of *M. tuberculosis*, geographical regions, and time periods (24). Thus, appropriate treatment for INH mono-resistant TB might prevent patients from progressing to MDR-TB (25–28). Conversely, approximately 15% of people with INH-susceptible (INH-S) but RIF-R TB are wrongly treated for MDR-TB in some settings (29, 30). These reports suggest that current diagnostic algorithms, which rely on RIF-R alone to diagnose MDR-TB, may need to be updated, especially where rapid testing for INH-R is available. Concurrent RIF and INH susceptibility testing is available using the Roche COBAS MTB and MTB-RIF/INH assay GenoType MTBDR*plus*, Abbott RealTime MTB RIF/INH, and BD Max MDR-TB assays (31–34); however, these tests must be performed in centralized laboratories because they require specialized facilities and highly trained staff (33, 35, 36), resulting in increased turnaround time.

Several studies have demonstrated that mutations in the promoter region of the *inhA* and the coding regions of the *fabG1* and *katG* genes are good predictors of INH-R (17, 37–40). These genes were targeted as part of the Xpert MTB/XDR (XDR) assay (Cepheid, USA), one of the first near-patient, rapid molecular assays to detect INH-R (as well as resistance to several other second-line TB drugs) (41). The XDR assay also detects mutations in the *oxyR-ahpC* intergenic region to provide additional coverage of INH-R; however, evidence suggests that mutations in *oxyR-ahpC* do not substantially increase sensitivity for INH-R (42, 43). INH-R detection performance by the XDR assay was similar to the WHO recommended line probe assay MTBDR*plus* (Bruker-Hain, Germany) with a sensitivity of 94% (95% confidence interval [CI]: 92–96) vs. 93% (95% CI: 90–95) and specificity of 100% (95% CI: 94–100) for both assays (44). However, the XDR assay was designed to be used as a reflex test for patients already diagnosed with TB using either Xpert MTB/RIF or Ultra tests, which adds cost and complexity to detecting INH resistance. There is clearly a gap between the WHO recommendation that all TB patients should receive a primary test for both INH-R and RIF-R and what is currently practical.

Here, we provide the first description of the MDRmDx assay, a cartridge-based prototype test intended for near-patient use. The MDRmDx assay is similar to the Ultra assay in many respects, but it improves upon the Ultra assay by simultaneously identifying both INH and RIF resistance, including RIF-R caused by the rapidly emerging I491F mutation. We explore the analytical performance of the MDRmDx assay compared to the Ultra assay to detect *M. tuberculosis* and RIF-R and describe the results of inclusivity, exclusivity, and interference testing. Our comprehensive mutant panel challenge shows that the MDRmDx assay can detect a wide repertoire of RRDR mutations, including resistance-causing mutations that have been missed by the Ultra assay. Finally, we compare the diagnostic accuracy of the Ultra and MDRmDx assays in a retrospective study of over 200 well-characterized clinical sputum samples obtained from the Foundation of Innovative New Diagnostics (FIND) biobank (45). Our results indicate that the MDRmDx assay could be an important addition to the TB diagnostic toolkit.

## MATERIALS AND METHODS

### Cartridge composition

The MDRmDx assay is a modified version of the cartridge-based Ultra assay that uses the GeneXpert instrument (Cepheid, Sunnyvale, CA, USA). It contains a newly incorporated I491F sloppy molecular beacon (SMB) probe to accompany the four *rpoB* RRDR-specific SMB probes used in the Ultra assay for detecting RIF-R mutations in *M. tuberculosis*. The MDRmDx assay also contains probes and primers to amplify resistance-conferring regions of the *inhA* promoter, and *katG* and *fabG1* genes for INH-R detection. These probes and primers are similar to those used in the Xpert XDR assay (41). Like the Ultra assay, the MDRmDX assay contains a sample processing and PCR control (SPC) component, which consists of a TaqMan probe and primers that detect the *Bacillus atrophaeus* (previously known as *B. globigii*) spores as part of the in-cartridge reagents. Similar to the Ultra assay, the MDRmDx assay detects the presence of *M. tuberculosis* using both a TaqMan probe and a molecular beacon, which target the *M. tuberculosis* multicopy *IS6110* and *IS1081* genes, respectively, along with their corresponding PCR primers. Except for the *IS6110*- and *IS1081*-specific probes, which are labeled with 6-carboxyfluorescein (FAM), each of the other probes is labeled with a different fluorophore to facilitate specific signal detection while minimizing optical crosstalk in a 10-color multiplex assay. Each MDRmDx cartridge contains all the PCR constituents in different lyophilized reagent beads, which are reconstituted and used in PCR using proprietary automated microfluidics defined by a specific assay definition format in the GeneXpert instrument (Cepheid, Sunnyvale, CA, USA).

### Sample testing procedures

When testing both Ultra and MDRmDx assays, each sample (sputum or buffer A containing Tris, EDTA, and tween, spiked with known numbers of *M. tuberculosis* CFU, clinical sputum sample, or cultured bacterial suspension) was mixed at a 2:1 ratio with sample reagent (SR) and incubated at room temperature for 15 min with occasional shaking (46). Two milliliters of the SR-sample mix were then added to the sample loading chamber of each cartridge. The loaded cartridges were placed into a GeneXpert instrument and run using an Assay Definition File (ADF), which defines the automated microfluidic processes for sample processing, DNA isolation, and PCR. For experiments using a DNA sample, 100 µL of the DNA sample diluted in water was loaded into the appropriate chamber of each cartridge, and the assay was run with an assay format (which does not include the sample processing steps) for directly testing genomic DNA.

### Assay result output

The MDRmDx assay was developed as an automated cartridge-based test, which combines sample processing and a two-step PCR amplification, the first phase being a symmetrical amplification, followed by a second phase of nested asymmetrical PCR. The presence of *M. tuberculosis* is detected by the real-time PCR signals generated in the presence of the multi-copy *IS6110* and *IS1081* genes or the *inhA* promoter region, obtained during the second amplification phase. The GeneXpert Diagnostics assay format for the Ultra assay was modified for the MDRmDx assay to incorporate additional rules for automated MTB detection and simultaneous detection of RIF and INH susceptibility or resistance. A “trace” category similar to that used in the Ultra assay was designated for tests, which yielded positive *IS6110* and/or *IS1081* signals only, to identify samples with low counts of *M. tuberculosis* targets. All *M. tuberculosis*-positive samples that were not “trace” proceeded to a post-PCR melt analysis stage, which is also performed by the automated GeneXpert software (Cepheid, Sunnyvale, CA, USA). This software generates second derivative melt curves that identify a melting temperature (T_m_) for each probe by locating where the second derivative melt curve crosses the zero baseline. These T_m_ values are then used to identify the presence of either a wild-type (WT) or mutant amplicon sequence based on whether the T_m_ is located within one or more pre-specified T_m_ parameters (T_m_ windows) that are usually associated with the presence of either a WT sequence or of one of several potential drug resistance-associated mutant sequences within the relevant amplicon. Identifying an amplicon with a T_m_ falling into a T_m_ window associated with either INH-R or RIF-R usually identifies the *M. tuberculosis* bacilli within the sample being tested as resistant to the corresponding drug. Unlike the Ultra assay, the MDRmDx assay does not provide semi-quantitative categories when *M. tuberculosis* is detected, although it retains the “trace” category output. The possible result outputs associated with RIF-R detection and INH-R detection are “RESISTANCE DETECTED,” “RESISTANCE NOT DETECTED,” or “RESISTANCE INDETERMINATE,” depending on the T_m_ values obtained for the analytes associated with a resistance call for these two drugs. The assay also distinguishes low-level INH resistance from high-level resistance, depending on whether a mutation is identified by the *inhA* promoter or *fabG1* SMB probes versus the *katG* SMB probe, respectively, and generates a “Low INH RESISTANCE DETECTED” output where appropriate.

### Preparation of *M. tuberculosis* and *M. bovis* BCG culture stocks and determination of CFU

The *M. tuberculosis* strain mc^2^6230 (H37Rv ΔRD1 Δ*panCD*) (47) or *M. bovis* BCG was cultured by inoculating 1:100 in 20 mL of Difco Middlebrook 7H9 medium (BD Biosciences, San Jose, CA, USA) supplemented with 10% BBL Middlebrook oleic acid-albumin-dextrose-catalase (OADC) enrichment (BD Biosciences) and 0.05% Tween 80 (Sigma-Aldrich, St. Louis, MO, USA). Calcium pantothenate (Sigma-Aldrich) was added at 24 μg/mL for the mc^2^6230 strain. The strains were passaged twice and quantified by plating 10^−5^, 10^−6^, and 10^−7^ dilutions in triplicate on Middlebrook 7H10 plates supplemented with 10% OADC and 24 μg/mL calcium pantothenate; 500 μL aliquots of cultures were made and stored at −80°C until use. The colonies were visible in approximately 3 weeks when colony counting was performed.

### Analytical sensitivity testing of MDRmDx in comparison with Ultra

The analytical sensitivity and limit of detection (LoD) of the MDRmDx assay were tested by spiking quantified BCG or *M. tuberculosis* mc^2^6230 into *M. tuberculosis*-negative sputum (human sputum from Seracare Life Sciences, Milford, MA, USA). Before spiking the sputum with known quantities of BCG or *M. tuberculosis* cells, all sputum pools were screened using the Ultra assay to confirm that they did not contain any detectable levels of *M. tuberculosis* DNA. For the dynamic range study, we spiked known numbers of CFU in sputum in a specific dilution series from 10^6^ CFU/mL to 10^2^ CFU/mL and tested four replicates for each dilution. We used a non-spiked sputum sample as a negative control. To determine the LoD, we first performed range finding with several concentrations of BCG and mc^2^6230 at different levels within a 5× to 0.5× range of the approximate LoD, to determine a presumptive LoD. We then tested 21 replicates at different CFU concentrations, representing the presumptive LoD, three concentrations below, and at least one concentration above the presumptive LoD. The assay LoD was defined as the lowest number of CFU that, when spiked into 1 mL of sputum, would result in the detection of *M. tuberculosis* in 95% of the positive samples. As a comparator, the same spiked sputum aliquots were tested with the Ultra assay.

### Analytical specificity and interference

Non-tuberculosis mycobacteria (NTM) species were either purchased from the American Type Culture Collection (ATCC) or kindly provided by National Jewish Health (Denver, CO, USA). The NTMs were cultured and quantified in a similar manner to that described here for *M. tuberculosis,* with occasional adjustments in culture conditions as required by specific NTM species. NTMs were tested at a final concentration equivalent to 10^6^ to 10^7^ CFU/mL in buffer A (Tris, EDTA, and Tween). The NTM repertoire consisted of *Mycobacterium avium* subsp*. avium, Mycobacterium celatum, Mycobacterium chelonae, Mycobacterium gordonae 14470, Mycobacterium gordonae 35760, Mycobacterium haemophilum, Mycobacterium abscessus, Mycobacterium asiaticum, Mycobacterium flavescens, Mycobacterium fortuitum, Mycobacterium gastri, Mycobacterium genavense, Mycobacterium intracellulare, Mycobacterium kansasii, Mycobacterium malmoense, Mycobacterium marinum, Mycobacterium scrofulaceum, Mycobacterium simiae, Mycobacterium szulgai, Mycobacterium thermoresistibile, Mycobacterium triviale, Mycobacterium vaccae, Mycobacterium xenopi, Mycobacterium smegmatis, Mycobacterium interjectum, Mycobacterium peregrinum, Mycobacterium mucogenicum, Mycobacterium goodie, Mycobacterium shimodei, Mycobacterium phlei, Mycobacterium terrae,* and *Mycobacterium chimaera. M. bovis* BCG (cells and DNA) at 3× LoD was used as positive controls, with tests containing only buffer A used as negative controls. We used 10^6^ genome equivalents of DNA for mycobacterial strains whose culture was unavailable. A selection of gram-positive and gram-negative bacteria that are known to colonize or infect the human respiratory tract and cause pulmonary symptoms similar to tuberculosis was also tested with the MDRmDx assay. The bacteria tested included *Klebsiella pneumoniae, Staphylococcus aureus, Streptococcus pyogenes, Streptococcus viridans, Staphylococcus epidermidis, Nocardia asteroides, Escherichia coli, Streptococcus agalactiae, Streptococcus pneumoniae, and Haemophilus influenzae*. We also tested six different species of *Candida* commonly found in human infections, like *Candida albicans*, *Candida auris*, *Candida krusei*, *Candida glabrata*, *Candida parapsilosis,* and *Candida tropicalis,* with the MDRmDx assay. To identify potential PCR interference to *M. tuberculosis* and drug resistance detection, eight clinically relevant NTM species (*M. marinum*, *M. gordonae 14470, M. gordonae 35760, M. intracellulare, M. kansasii, Mycobacterium avium* subsp*. avium*, *M. kansasii*, and *M. abscessus*) mixed with *M. bovis* BCG were tested. A concentration of 10^6^ NTM CFU/mL was mixed with *M. bovis* BCG at 3× the MDRmDx assay LoD, and the mixed samples were then tested for the presence of tuberculosis in replicates of three. Buffer A was used as the negative control, and *M. bovis* BCG at 3× LoD concentration was used as the positive control. The bacterial suspensions were processed and loaded into the cartridge as previously described.

### Inclusivity testing and mutation panel challenge

A panel of clinical isolates belonging to seven different *M. tuberculosis* lineages with varying *IS6110* copy numbers and *Mycobacterium tuberculosis* complex strains *M. bovis, M. africanum, M. canetti*, *and M. microti* was tested at a concentration that was approximately 3× of a predetermined *M. bovis* BCG DNA LoD. Buffer A was used as a negative control. Each isolate was tested in replicates of three and run on a GeneXpert instrument.

The mutation panel challenge was performed using *M. tuberculosis* DNA from the Tropical Disease Research (TDR) Strain Bank, the FIND, and the International Tuberculosis Research Center (Masan, South Korea). The DNA was quantified using a Qubit double-stranded DNA (dsDNA) HS assay kit. We tested all the RIF-R causing mutations listed in the 2023 WHO mutation catalog (17) that had a reported global at least 0.5% prevalence among RIF-R isolates. Additionally, we investigated certain RIF resistance mutations in the *rpoB* gene that have been reported to be missed by the Ultra assay, including the non-RRDR mutation I491F (Table 1), and incorporated several other strains in the panel with mutations across the RRDR region (Table S1). We also analyzed a comprehensive panel of clinically significant INH-R mutations with the MDRmDx assay (Table S2).

**TABLE 1:** Mutations in the *rpoB* RRDR that were missed by Ultra but detected by MDRmDx^*a*^

Mutation	Ultra result output	MDRmDx result output
I491F*	MTB DETECTED LOW; **RIF Resistance NOT DETECTED**	MTB DETECTED; **RIF Resistance DETECTED**; INH Resistance NOT DETECTED
a1296g (Q513Q)	MTB DETECTED LOW; **RIF Resistance INDETERMINATE**	MTB DETECTED; **RIF Resistance NOT DETECTED**; INH Resistance NOT DETECTED
Q432L	MTB DETECTED LOW; **RIF Resistance INDETERMINATE**	MTB DETECTED; **RIF Resistance DETECTED**; INH Resistance DETECTED
Q432P	MTB DETECTED LOW; **RIF Resistance INDETERMINATE**	MTB DETECTED; **RIF Resistance DETECTED**; INH Resistance DETECTED
D435G*	MTB DETECTED LOW; **RIF Resistance NOT DETECTED**	MTB DETECTED; **RIF Resistance DETECTED**; INH Resistance NOT DETECTED
D435G*+N437H	MTB DETECTED LOW; **RIF Resistance INDETERMINATE**	MTB DETECTED; **RIF Resistance DETECTED**; Low INH Resistance DETECTED
D435Y*+N437H	MTB DETECTED LOW; **RIF Resistance INDETERMINATE**	MTB DETECTED; **RIF Resistance DETECTED**; INH Resistance DETECTED
S431T+M434I+H445N	MTB DETECTED LOW; **RIF Resistance INDETERMINATE**	MTB DETECTED; **RIF Resistance DETECTED**; INH Resistance DETECTED

^*a*^ An asterisk (*) indicates mutations with a prevalence of ≥0.5% as in the WHO 2023 mutation catalogue. Boldface type highlights differences in rifampicin resistance result output.

### Preparation of mixed cells to test for detection of heteroresistance

We evaluated the efficiency of the MDRmDx assay to detect heteroresistance by testing mixtures of quantified H37Rv mc^2^6230 cells and formalin-fixed cells of the *M. tuberculosis* MDR strain TDR-0074, which contains *rpoB* S450L and *katG* S315T mutations. To accurately determine the concentration of the fixed TDR-0074 cells, we tested serial dilutions of TDR-0074 stocks and quantified WT H37Rv mc^2^6230 cells using the Ultra assay and adjusted the nominal TDR-0074 CFU count so that similar CFU dilutions of both strains generated comparable cycle threshold (*C_T_*) values. After quantification, a series of H37Rv mc^2^6230:TDR-0074 strain mixtures containing a total of 10,000 cfu/mL per mixture were tested with varying proportions (0%, 5%, 10%, 20%, 25%, 50%, 75%, 90%, and 100%) of H37Rv mc^2^6230 using both the Ultra and MDRmDx assays.

We also compared the ability of the MDRmDx and XDR assays to detect isoniazid heteroresistance using quantified preparations of hardened *E. coli* cells (Maine Molecular Quality Controls Inc., Saco, ME, USA) that had been transfected with plasmids containing WT or mutant target sequences, including C(–15)T in the *inhA* promoter, S513T in the *katG* gene, and L203L in *fabG1* (MMQCI WT and mutant constructs, respectively). As described above for the mixture studies, a total of 10,000 cells/mL containing MMQCI WT: MMQCI mutant mixtures were prepared for each mixture containing 0%, 10%, 20%, 25%, 30%, 35%, and 50% of mutant against the WT background. Three replicates of each mixture were tested simultaneously by both the MDRmDx and XDR assays.

### Clinical study protocol

We performed a blinded study of the MDRmDx assay using banked clinical sputum samples to compare the diagnostic accuracy of the MDRmDx versus the Ultra assay to detect *M. tuberculosis* and resistance to rifampicin and/or isoniazid. MGIT liquid culture and MGIT-based P-DST were used as the clinical reference standard. Sanger DNA sequencing of the assay targets was used to provide a final resistance reference determination for discordances where the MDRmDx and/or the Ultra assays identified resistance, but the MGIT-based P-DST generated a drug-susceptible result. However, sequencing was not used to provide a final resistance determination when the MGIT-based P-DST generated a drug-resistant result, but the MDRmDx and/or Ultra assay produced a drug-susceptible result because not all the genetic causes of INH-R or RIF-R are known. Accordingly, we provide Sanger sequence-adjusted estimates of specificity but not sensitivity.

The FIND sputum bank (45) provided us with 220 frozen sputum samples for which smear microscopy status, MTB culture status, and P-DST for INH and RIF had been determined previously. Four groups of specimens were selected for the evaluation, consisting of 55 samples that were (i) culture positive/smear positive (C+/S+), (ii) 55 that were culture positive/smear negative (C+/S−), and (iii) 55 that were specifically chosen because they were C+/S+ and RIF-R and/or INH-R. Some isolates in the C+/S+ and the C+/S− groups were also tested by P-DST for RIF and INH susceptibility. These P-DST tested samples were included in the analysis of RIF-R and INH-R detection performance. An additional group (iv) of 55 who were culture negative/smear negative (C−/S−) was included in the study for specificity calculations. All samples were stored at −80 °C and were not reported to have undergone any freeze-thaw cycles. Each sputum sample was expected to be provided as triplicate 0.5 mL aliquots; however, some samples were found to have inadequate volumes upon receipt. Sputum sample aliquots were allowed to thaw at room temperature, after which each of the three aliquots from the same original sputum sample was pooled together into a single 50 mL polypropylene tube. The volume of each sputum sample in a tube was measured by pipette, and then SR at 2× the volume of the measured sputum was added to each tube, resulting in a final SR:sputum ratio of 2:1. Each tube was then gently shaken (10×) and incubated for 10 min at room temperature. After incubation, the tube was again gently shaken (10×), followed by 5 min incubation at room temperature. For each SR-sputum mix, 2 mL was transferred to a MDRmDx cartridge, and 2 mL was transferred to an Ultra cartridge for testing using a GeneXpert instrument. The order in which the SR-sputum was placed in the MDRmDx versus the Ultra cartridges was randomized. Testing was performed in a blinded manner; the results were analyzed only after all the runs were completed, and the results were locked. A primary analysis was performed to directly compare the performance of the MDRmDX versus the Ultra assay on the same set of samples. For the primary analysis, SR-sputum samples with volumes below (<4 mL) what was needed to perform both Ultra and the MDRmDx tests, or samples where one or both of the assays could not be performed due to “ERROR” messages, were excluded. A pre-planned secondary analysis then separately calculated the performance of each assay using all samples that were tested by that assay compared with the clinical reference standard (and reference standard incorporating Sanger sequencing where indicated).

### Statistical analysis

LoDs were calculated by using Probit analysis on R version 4.4.1 using ggplot2 version 3.5.1. For the analysis, the percentages of the replicates resulting in successful MTB detection and drug-susceptibility calls at each input CFU concentration in sputum were used for both the MDRmDx and Ultra assays. This calculation was derived from the fitted probit model of the concentration, where the estimated detection probability was 95%. The 95% CI for the estimated LoD was determined from the concentrations representing the upper and lower LoD ranges, as calculated by the probit regression model.

Clinical data were analyzed using SAS 9.4. Sensitivity and specificity were estimated using simple proportions and 95% CIs using the Wilson score method. For the MDRmDx assay, results with “Error” (codes 2125 and 5011) and “Invalid” outputs were classified as “non-determinate” for *M. tuberculosis* detection and excluded from primary sensitivity and specificity calculations. With respect to the detection of drug resistance, the MDRmDx assay “indeterminate” results for RIF and INH resistance were similarly excluded from sensitivity and specificity calculations. Because we were interested in population averages, we used a marginal model approach to estimating differences between MDRmDx and Ultra assays for sensitivity or specificity using logistic regression fit using generalized estimating equations (GEE) to account for correlation between results from the same participant. When the results were either all positive or all negative, logistic regression analysis could not be done. All *P*-values are two-sided, and testing was done at a 5% significance level.

## RESULTS

### Analytic LoD

**Fig 1:**
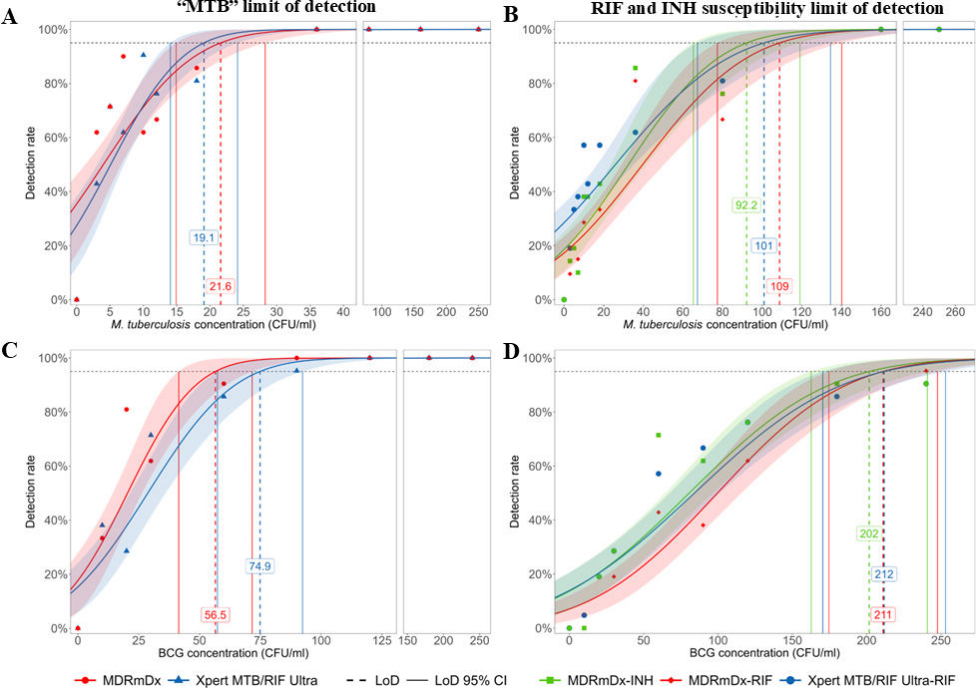
Assay limit of detection. Limits of detection (LoD) for the MDRmDx assay versus the Ultra assay tested with *M. tuberculosis* strain H37Rv mc^2^6230 (**A and B**) or *M. bovis* strain BCG (**C and D**). Panels A and C show limits of detection for CFU of the indicated strains, with red lines indicating MDRmDx assay performance and blue lines indicating Ultra assay performance. Panels B and D show LoD for RIF and INH susceptibility detection with the red lines indicating MDRmDx assay RIF-S performance, blue lines indicating Ultra assay RIF-S performance, and green lines indicating MDRmDx assay INH-S performance. Individual data points are also shown using the same colors as the lines to identify data generated with each assay or drug susceptibility test.

LoD studies were performed on both MDRmDx and Ultra assays using the same aliquots of quantified test bacteria. Serial dilutions of quantified *M. tuberculosis* H37Rv mc^2^6230 (H37Rv) and BCG CFU were spiked into TB-negative sputum in two separate experiments. For testing *M. tuberculosis* H37Rv, 10 concentrations (3, 5, 7, 10, 12, 18, 36, 80, 160, and 250 CFU/mL) of this strain were tested with 21 replicates per concentration. The LoD for detecting *M. tuberculosis* in sputum was 21.6 CFU/mL (95% CI: 14.9–28.3) for the MDRmDx assay versus 19.1 CFU/mL (95% CI: 14.0–24.1) for the Ultra assay (Fig. 1A). The LoD for detecting rifampicin susceptibility (as opposed to RIF-R, which is dependent on the specific *rpoB* mutation present in each *M. tuberculosis* strain) was 108.8 CFU/mL (95% CI: 77.4–140.2) for the MDRmDx assay versus 100.9 CFU/mL (95% CI: 67.3–134.5) for the Ultra assay (Fig. 1B). Both assays were able to detect *M. tuberculosis* and RIF-S 100% of the time, down to concentrations of 160 CFU/mL. It is worth noting that the MDRmDx produced comparable results to the Ultra assay, although it is more complex. The MDRmDx assay also includes three targets to detect INH-S (as opposed to INH-R, which can be detected by mutations present in any one of the three targets, when *M. tuberculosis* is detected). The INH-S detection LoD for *M. tuberculosis* was 92.2 CFU/mL (95% CI: 65.3–119.1) (Fig. 1B). This LoD is comparable to the *M. tuberculosis* INH-S LoD of 79.8 CFU/mL previously reported for the Xpert XDR assay (41).

*M. tuberculosis* H37Rv mc^2^6230 contains 17 *IS6110* and four *IS1081* elements that are targeted in the *M. tuberculosis* identification portion of both assays. Since the number of these elements can vary across different *M. tuberculosis* strains, we performed similar LoD studies using BCG CFU as the challenge strain since BCG contains only one copy of *IS6110* and five copies of *IS1081* (10). Eight concentrations of BCG (10, 20, 30, 60, 90, 120, 180, and 240 CFU/mL) were spiked into TB-negative sputum and tested in parallel with the Ultra assay in 21 replicates per concentration. The LoD for detecting BCG in sputum was 56.5 CFU/mL (95% CI: 41.5–71.6 CFU/mL) for the MDRmDx assay versus 74.9 CFU/mL (95% CI: 57.4–92.4) for the Ultra assay (Fig. 1C). The LoD for RIF-S detection was 211.1 CFU/mL (95% CI: 174.6–247.6) for the MDRmDx assay versus 211.8 CFU/mL for the Ultra assay (95% CI: 170.5–253.1) (Fig. 1D). The INH-S detection LoD for BCG was 201.8 CFU/mL (95% CI: 162.7–240.9) for the MDRmDx assay (Fig. 1D). The difference between the *M. tuberculosis* and BCG TB detection LoDs that we established for both assays can be understood in the context of *M. tuberculosis* detection since the Ultra assay is known to be less sensitive for detecting BCG due to its lower copy number of *IS6110* elements (43).

### Mutant panel challenge

**Fig 2:**
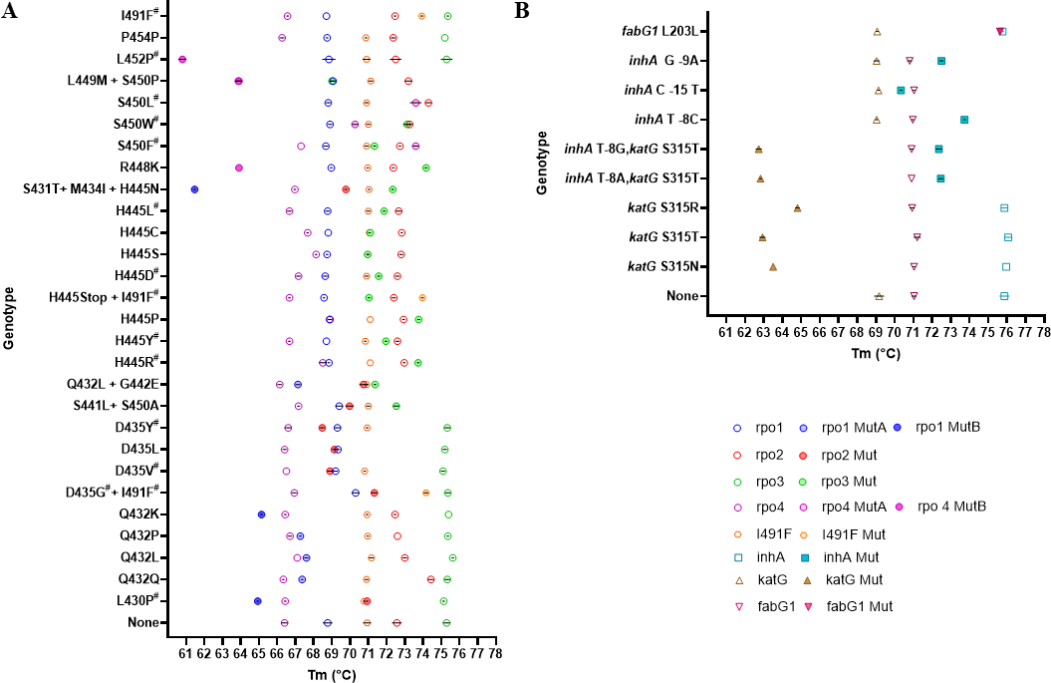
Probe Tm values in the presence of mutant and WT DNA. (**A**) Tm values of *rpoB* probes used in the MDRmDx assay are shown in tests against a panel of RIF-R associated *rpoB* mutant DNA. The mean Tm value ± SD with error (represented by the black line horizontal to the x-axis) was determined for the indicated RIF-R associated *rpoB* mutations as detected by MDRmDx assay. Three replicates were tested for each sample of DNA. A hashtag (#) indicates mutations with a prevalence of ≥0.5% as in the WHO 2023 catalog. None = WT DNA tested. (**B**) MDRmDx assay was tested against a panel of INH-R mutant strains. The mean Tm value ± SD with error (represented by a black line horizontal to the x-axis) was graphed for INH-R associated mutations.

**Fig 3:**
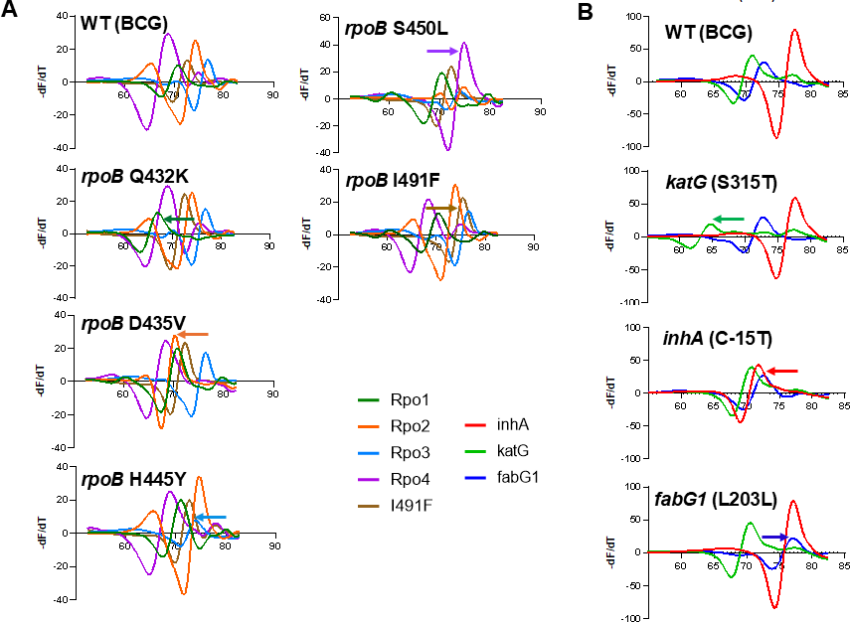
Mutation detection using melting temperature curve analysis. (**A**) Detection of *M. bovis* BCG and *M. tuberculosis rpoB* gene mutations associated with RIF-R. Second derivative melting temperature (Tm) curves of five *rpoB*-specific sloppy molecular beacon (SMB) probes (*rpo1, rpo2, rpo3, rpo4,* and *I491F*) are shown. Each SMB is indicated by a different colored line. The point where a Tm curve intersects with the -dF/dT baseline identifies the exact Tm of a particular SMB to the indicated wild-type (WT) or mutant *rpoB* target. WT (BCG) does not contain any mutations in these DNA regions, and a Tm analysis of WT BCG DNA is shown as a WT control to identify Tm curves indicative of RIF-S. A similar Tm analysis is also shown using DNA derived from five different clinical *M. tuberculosis* strains, each containing a different RIF-R associated *rpoB* mutation that is targeted by one of the five SMBs tested. The Tm shifts from WT to mutant values for each SMB (identifying a sample as an *rpoB* and RIF-R mutant) and the *rpoB* mutation present in the tested DNA is also indicated. (**B**) Detection of *M. tuberculosis* gene mutations associated with INH-R. Three SMB probes designed to identify the most common mutations associated with INH-R [*inhA* C(–15)T, *katG* (S315T), and *fabG1* (L203L)] were tested. WT (BCG) does not contain any mutations at these alleles, and a Tm analysis of WT BCG DNA is shown as a WT control and identify T_m_ curves indicative of INH-S. The Tm shift from WT to mutant values for each SMB (identifying a sample as an INH-R mutant), and the mutation present in the tested DNA is also indicated.

We evaluated the ability of the MDRmDx assay to detect drug-resistance-associated mutations in clinical *M. tuberculosis* isolates. For RIF-R determination, we tested a panel of RIF-R clinical isolates representing all *rpoB* gene mutants present at global frequencies of ≥0.5% as reported to be associated with RIF resistance (WHO mutation catalog for *M. tuberculosis* 2023) (Fig. 2A; Table S1), except for the *rpoB* V170F mutation (17, 48), which was not included in the MDRmDx assay for technical reasons. For INH-R, we tested DNA extracted from a panel of 9 INH-R clinical strains. The MDRmDx assay correctly detected INH resistance-conferring mutations in the *inhA* promoter region [*inhA* C(−15)T, *inhA* G(-9)A, *inhA* T(−8)A, *inhA* T(−8)G, and *inhA* T(−8)C], in codon 315 of the *katG* gene (S315T, S315N, and S315R), and in the *fabG1* L203L as INH-R (Fig. 2B; Table S2). Each mutation type was tested using quantified genomic DNA at 3× the LoD we established for detecting WT DNA as RIF-S and INH-S. If tests of a RIF-R strain did not produce the expected resistance call at 3× of the WT DNA LoD, it was tested again using 6× and if needed, 10× of the WT DNA LoD. Variation in the LoD among resistant samples is not entirely unexpected, since depending on the mutation, the corresponding mutant LoD may be worse than the WT LoD due to probe destabilization by that specific mutation and hence reduction in analytical sensitivity as compared to the WT sequence (RIF-S). Most mutations associated with RIF-R and INH-R were detected by both assays, as indicated by a shift of one or more SMB probes from the expected WT Tm value to a value expected to be produced by a mutation in the region targeted by that probe (Fig. 3A and B). RIF-R mutations that were correctly detected in the MDRmDx but either incorrectly detected or detected as “indeterminate” by Ultra are shown in Table 1, with the Tm values generated by wild-type RIF-S, and all tested RIF-R mutations are shown in Table S1. The results of MDRmDx testing of INH-R isolates are shown in Table S2. Our results showed that the highly prevalent MDR *katG* S315T and *rpoB* S450L double mutants were easily detected as “RIF Resistance Detected” and “INH Resistance Detected” at 3× the WT LoD. All other RIF-R conferring mutations present at ≥0.5% global frequencies were also identified as RIF-R at between 3× and 10× the LoDs for RIF-S (Fig. 2A; Table S1), including *rpoB* I491F. Two silent mutations, Q432Q and P454P (which are not associated with RIF-R), were identified as “RIF Resistance Not Detected” by the MDRmDx assay (Fig. 2A; Table S1), whereas the Ultra assay detected only the P454P mutation as “RIF Resistance Not Detected” and designated the Q432Q mutation as “RIF Resistance Indeterminate” (Table 1). Other low-frequency *rpoB* mutations, which are known to cause RIF-R but generate “RIF Resistance Indeterminate” results using the Ultra assay (49), were tested by both the MDRmDx and Ultra assays. The mutations Q432L and Q432P were called “RIF Resistance Indeterminate” by Ultra, as expected but were correctly identified as RIF-R by the MDRmDx assay (Table 1). In addition, the D435G mutation (present at a global frequency of 0.9% among RIF-R strains), which was incorrectly identified by Ultra as RIF-S, was correctly detected as RIF-R by the MDRmDx assay. The MDRmDx assay also clearly identified *M. tuberculosis* strains encoding three different mutations in proximity to each other in the RRDR (S431T+M434I+H445N) as RIF-R, while this same triple mutant led to a “RIF Resistance Indeterminate” result when tested by the Ultra assay (Table 1) (10).

### Detection of heteroresistance

**Fig 4:**
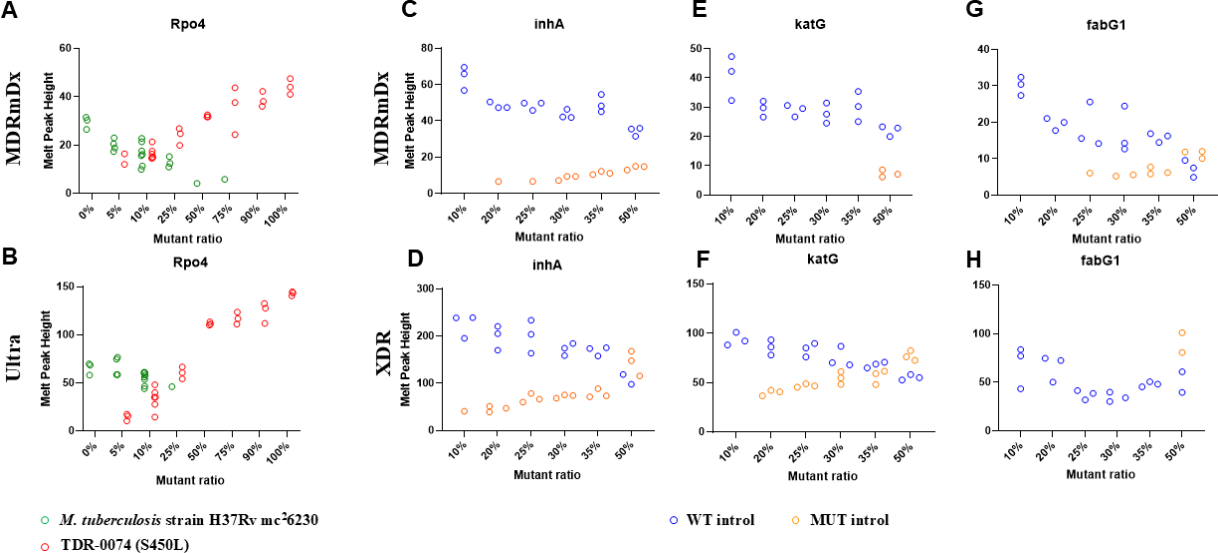
Heteroresistance detection. (**A and B**) Detection of the RIF-R associated *rpoB* S450L mutation in mixtures of WT *M. tuberculosis* strain H37Rv and mutant *M. tuberculosis* strain TDR-0074 cells. Different ratios of mutant and wild-type cells, keeping the total number of cells constant, were tested with both the MDRmDx (**A**) and the Ultra (**B**) assays. The height of the Tm peaks of the *rpo4* probe (which can differentiate between the WT *rpoB* sequence and the S450L *rpoB* mutation present in TDR-0074) in these cell mixtures is indicated on the Y axis with green and red open circles showing WT and mutant Tm peaks, respectively. The results of three or more replicates tested for each mutant ratio mixture are shown. (**C–H**) Detection of INH-R associated [*inhA* C(–15)T, *katG* (S315T), and *fabG1* (L203L)] mutations in mixtures of WT INTROLs, which contain cloned WT sequences for these genes and mutant INTROLs, which contain the cloned INH-R associated mutations indicated. Different ratios of mutant and wild-type INTROLs, keeping the total number of cells constant, were tested with both the MDRmDx (**C, E, G**) and the XDR (**D, F, H**) assays. The Tm peak heights of the SMB, which differentiates between WT and mutant *inhA, katG,* or *fabG1* alleles, are indicated on the Y axis, with blue and orange open circles showing WT and mutant Tm peaks, respectively. The results of three replicates tested for each mutant ratio mixture are shown. The Y axis of the dot plot was jittered slightly when dots would otherwise appear on top of each other.

Persons with TB can sometimes have a mixture of drug-susceptible and drug-resistant *M. tuberculosis* in their sputum, referred to as heteroresistance (50, 51). The Ultra assay can detect between 10% and 40% of RIF-R mixed with RIF-S, and the XDR assay can detect 15% and 20% of INH-R mixed with INH-S, depending on the specific mutation (10, 41). We tested the ability of the MDRmDx assay to detect heteroresistance in head-to-head comparisons with the Ultra assay for RIF-R and the XDR assay for INH-R, respectively. For RIF heteroresistance, we tested mixtures of WT H37Rv cells and pre-quantitated formalin-fixed *M. tuberculosis* cells from strain TDR-0074, an MDR strain that contained both *rpoB* S450L and *katG* S315T mutations. We created a series of mixtures using these two strains containing 5%, 10%, 25%, 50%, 75%, 90%, and 100% of resistant *M. tuberculosis* cells mixed with WT cells, with each dilution having the same total number of (resistant + WT) cells. For the *rpoB* S450L mutation, the *rpo4* SMB probe was designed to yield a higher T_m_ in the presence of the RIF-R mutant allele (which would result in a “RIF Resistance Detected” call) compared to the WT allele (which would produce a “RIF Resistance Not Detected” call). We tested three replicates for most of the cell mixtures except for those with lower proportions of mutant cells, where we ran between 4 and 8 replicates. With this high T_m_ mutation, we found that both the MDRmDx (Fig. 4A) and Ultra (Fig. 4B) assays were able to detect mutant T_m_ values in mixtures containing as little as 5% mutant cells (in two of the four replicates for MDRmDx and in three out of four replicates for Ultra). The MDRmDx assay detected RIF-R in six out of seven replicates (one error) while the Ultra detected RIF-R in six out of eight replicates for mixtures with as little as 10% mutant cells. Mixtures with greater than or equal to 25% RIF-R mutant cells generated a RIF-R call in all replicates for both the assays, with the height of the elevated T_m_ peak (mutant) for the probe *rpo4* increasing with the increasing proportion of mutant cells present in the mixture. Similarly, our results showed that the *katG* S315T mutation was detected as INH-R by the MDRmDx assay in two out of three replicates in mixtures containing as little as 25% mutant.

To test the ability of all three different INH-R SMB probes to detect heteroresistance, we obtained a recombinant target INTROL TB XDR Mut (panel M345) (Maine Molecular Quality Controls, Inc. Saco, ME, USA), consisting of hardened *E. coli* cells transformed with single copies of portions of the *M. tuberculosis inhA*, *katG,* and *fabG1* genes encoding the INH-R mutations *inhA* C(−15)T, *katG* S315T, and *fabG1* L203L. We also obtained INTROL TB-XDR WT, which has a design similar to the XDR Mut INTROL except that it contains only WT gene sequences. XDR Mut and WT INTROL cell mixtures containing 0%, 10%, 20%, 25%, 30%, 35%, and 50% of the mutant INTROL cells against a background of the TB-WT cells (prepared to result in the same number of total INTROL cells in each mixture) were tested using both MDRmDx and XDR assays. Although the *fabG1* probe was designed to generate a higher T_m_ value in the presence of L203L mutation, the *inhA* and *katG* probes were designed to generate lower T_m_ values in the presence of either C(−15)T or S315T mutations. We found that the MDRmDx assay detected INH-R *fabG1* mutations better than the XDR assay, INH-R *inhA* mutations were approximately equivalent to the XDR assay, and INH-R *katG* mutations were less well detected than the XDR assay. For the *inhA* promoter, the MDRmDx assay detected resistance in one out of three and three out of three of the replicates containing 20%–25% and ≥30% mutant (Fig. 4C), respectively, versus the XDR assay which detected resistance in one out of three and three out of three of the replicates containing 10% and ≥20% mutant (Fig. 4D), respectively. For *katG*, the MDRmDx assay detected resistance in all the replicates containing ≥50% mutant (Fig. 4E) versus the XDR assay, which detected resistance in all the replicates containing ≥20% mutant (Fig. 4F). For *fabG1*, the MDRmDx assay detected resistance in one out of three, two out of three, and three out of three replicates containing 25%, 30%, and 35% of mutant (Fig. 4G), respectively, versus the XDR assay, which detected no resistance until the sample contained 50% *fabG1* mutants (Fig. 4H). This observation contrasted with our earlier study (described above) using the *katG* S315T mutant *M. tuberculosis* TDR 0074 strain mixed with WT *M. tuberculosis* cells, where the MDRmDx assay detected INH heteroresistance at comparable levels to XDR assay (41).

### Assay inclusivity

We examined a variety of clinical *M. tuberculosis* strains with different numbers of *IS6110* copies belonging to the seven different *M. tuberculosis* lineages (Table S3A), as well as four *Mycobacterium tuberculosis* Complex (MTBC) strains (Table S3B). Genomic DNA extracted from each strain was tested in three replicates at a concentration of 3× or more of the estimated LoD for WT H37Rv mc^2^6230. The MDRmDx assay correctly detected the presence of *M. tuberculosis* in all tests and generated correct “Resistance Not Detected” calls for both RIF and INH as expected.

### Analytical specificity and NTM interference

We tested whether the MDRmDx assay showed any cross-reactivity with 31 clinically relevant NTM species (Table S4A), 10 gram-positive and gram-negative bacteria found commonly in oral and sputum flora (Table S4B) and six different *Candida* species (Table S4C). Each species was evaluated at 10^6^ CFU to 10^7^ CFU or 10^6^ genome copies per reaction in triplicates. Most of the NTMs generated *rpo2* probe real-time signals with an average *C_T_* ranging from 17 to 40 (Fig. S1) and *Mycobacterium chimaera* produced an additional signal from the *rpo1* probe. However, none of the NTMs produced signals from the *M. tuberculosis* specific-*IS6110/IS1081* probes, and the MDRmDx assay produced a result output of “MTB not detected” for all the NTMs. The assay also did not generate any T_m_ measurements for the RIF-R and INH-R probes due to automatic termination of the melt stage in the absence of *IS6110-IS1081* and *inhA* promoter real-time signals. To test for the potential of NTM present in a sample to interfere with the detection of *M. tuberculosis* or interfere with the detection of drug resistance in case of a co-infection, we mixed a large excess (10^6^ CFU/mL) of selected NTMs (*M. marinum*, *M. gordonae*, *M. avium*, *M. intracellulare*, *M. chimaera*, *M. kansasii*, and *M. abscessus*) with BCG at 3× the LoD for *M. tuberculosis* detection (Table S5). All the NTM-BCG mixtures generated the correct “MTB detected, RIF resistance not detected; INH resistance not detected” output indicating that even NTM-BCG mixtures containing high numbers of NTM cells and low numbers of BCG would not lead to false resistance detection results.

### Dynamic range comparison with Ultra

A range of concentrations from 10^6^ CFU/mL to 100 CFU/mL of BCG and *M. tuberculosis* H37Rv mc^2^6230 were separately spiked into sputum to determine the dynamic range of the MDRmDx assay in comparison to the Ultra assay in two independent experiments. The lowest *C_T_* value generated from among the four *rpoB* probes for each assay was plotted against the log CFU/mL for each test concentration of either *M. tuberculosis* (Fig. S2A) or BCG (Fig. S2B). The *rpoB* probe values resulted in a linear relationship to the CFU/mL spiked in sputum for the MDRmDX assay similar to that seen with the Ultra assay and a linear regression analysis produced comparable R-squared values for both assays (Ultra = 0.91 and MDRmDx = 0.91 for mc^2^6230; and Ultra = 0.91 and MDRmDx = 0.89 for BCG) This indicated that even though the semi-quantitative call-out is absent in the MDRmDx assay, the earliest *rpoB* probe *C_T_* can still serve as a semi-quantitative measure of the number of bacilli in the sample, which is expected to be comparable to the semi-quantitation outputs provided by the Ultra assay.

### Retrospective study of clinical sputum samples: primary analysis

We compared the clinical diagnostic accuracy of the MDRmDX and Ultra assays using 220 frozen sputum samples from confirmed TB patients and controls obtained from the FIND specimen bank (45). The different sample categories, workflow for the clinical study, and the result output of the investigational assay are shown in Fig. S3 to S5. Participant characteristics are shown on Table S6. Notably, only 9/165 (5.5%) of the C+ samples were collected within 5 years before testing by our assays, whereas 59/165 (35.8%) of the C+ samples were collected 7–8 years before testing and an additional 97/165 (58.8%) were collected 9–12 years before testing. We employed two different approaches to analyze the comparative performance between the MDRmDx and Ultra assays. The primary analysis is described below, and the secondary analysis is shown in detail in the supplemental material (see Table S7 and Retrospective study of clinical sputum samples: Secondary analysis).

Our primary analysis was designed to obtain an accurate comparison of each assay without variability due to test sputum sample volume, length of storage, or *M. tuberculosis* bacillary load. Thus, the primary analysis included only samples that had been tested by both MDRmDx and Ultra assays and yielded a valid result for both assays. Twenty-two of the 220 (10%) study samples were excluded from this analysis. Four excluded samples had volumes that were too low for testing with both assays and were tested only with the MDRmDx assay for inclusion in the secondary analysis. Five samples tested with the MDRmDx assay and 10 samples tested with the Ultra assay produced “error” or “invalid” results and were excluded from the primary analysis. One additional sample produced “error” in both the MDRmDx and Ultra assays and was also excluded from analysis. Finally, two samples were excluded from analysis due to a labeling error which resulted in one sample being tested twice only with the MDRmDx assay and not on the Ultra assay and a second sample being tested twice with the Ultra assay but not on the MDRmDx assay (Fig. S3).

In the primary analysis of the remaining 198 samples, the sensitivity and specificity of MDRmDx assay and Ultra were individually calculated by comparing their results with the MGIT culture-based reference standards. Combining results from sampling groups, the diagnostic sensitivities for MTB detection were 136/149 (91.3%, 95% CI: 85.6–94.8) and 139/149 (93.3%, 95% CI: 88.1–96.3) for the MDRmDx and Ultra assays, respectively; the difference in sensitivity was −2.0% (95% CI: −4.3, 0.2; *P* = 0.08). The diagnostic specificities were 48/49 (98.0%, 95% CI: 89.3-99.6) for both the MDRmDx and Ultra assays. For C+/S+ sputum, the sensitivity of MTB detection was 97/99 (98.0%, 95% CI: 92.9–99.4) for both MDRmDx and Ultra assays. For C+/S− sputum, the sensitivity for MTB detection was 39/50 (78.0%, 95% CI: 64.8–87.2) and 42/50 (84.0%, 95% CI: 71.5–91.7) for the MDRmDx and Ultra assays (Table 2), respectively; the difference in sensitivities was −6.0% (95% CI: −12.6, 0.6; *P* = 0.07). All three C+/S− samples detected by the Ultra assay but not by the MDRmDx assay produced an “MTB trace detected” semi-quantitative result by the Ultra assay and had been collected 3, 8, and 10 years before testing in our study.

**TABLE 2:** Sensitivity and unadjusted and sequence-adjusted specificity estimates for the primary analysis of the clinical sample study (when valid results were available for both MDRmDx and Ultra assays)^*a*^

	Assay/reference standard results^*b*^				Sensitivity		Specificity	
	+/+	+/−	−/+	−/−	%	95% CI	%	95% CI
MDRmDx assay								
MTB detection	136	1	13	48	91.3	85.6–94.8	98.0	89.3–99.6
Among S+	97	0	2	0	98.0	92.9–99.4	N/A	N/A
Among S−	39	1	11	48	78.0	64.8–87.2	N/A	N/A
RIF	75	3	0	45	100	95.1–100	93.8	83.2–97.9
Among S+	52	2	0	34	100	93.1–100	94.4	81.9–98.5
Among S−	23	1	0	11	100	85.7–100	91.7	64.6–98.5
RIF (sequence adjusted)	78	0	0	45	N/A	N/A	100	92.1-100
INH	76	0	1	38	98.7	93.0–99.8	100	90.8–100
Among S+	54	0	1	28	98.2	90.4–99.7	100	87.9–100
Among S−	22	0	0	10	100	85.1–100	100	72.2–100
Ultra assay								
MTB detection	139	1	10	48	93.3	88.1–96.3	98.0	89.3–99.6
Among S+	97	0	2	0	98.0	92.9–99.4	N/A	N/A
Among S−	42	1	8	48	84.0	71.5–91.7	N/A	N/A
RIF	73	3	0	43	100	95.0–100	93.5	82.5–97.8
Among S+	52	2	0	34	100	93.1–100	94.4	81.9–98.5
Among S−	21	1	0	9	100	84.5–100	90.0	59.6–98.2
RIF (sequence adjusted)	76	0	0	43	N/A	N/A	100	91.8–100

^*a*^ 95% CIs were calculated using Wilson score method. MTB, *Mycobacterium tuberculosis*; N/A, not applicable.

^*b*^ For MTB detection, + denotes MTB detected and − denotes MTB not detected. For drug resistance, + denotes resistance detected and − denotes no resistance detected. +/+, positive by both assay and reference standard; +/−, positive by assay and negative by reference standard; −/+, negative by assay and positive by reference standard; −/−, negative by both assay and reference standard.

Of the 198 samples included in the primary analysis, 149 were C+ (S+ or S−), and 125 of these had RIF P-DST results available and had *M. tuberculosis* detected by both MDRmDx and Ultra assays, of which 76 were RIF-R and 49 were RIF-S (Fig. S4). The sensitivity for RIF-R detection was 75/75 (100%, 95% CI: 95.1–100) with one RIF indeterminate sample for the MDRmDx assay and 73/73 (100%, 95% CI: 95.0–100) with three indeterminate samples for the Ultra assay (Table 2). The specificity for RIF-R detection was 45/48 (93.8%, 95% CI: 83.2–97.9) with one indeterminate for the MDRmDX assay and 43/46 (93.5%, 95% CI: 82.5–97.8) with three indeterminates for the Ultra assay; the difference in specificity was 0.3% (95% CI: −0.2, 0.7; *P* = 0.26) (Table 2). Specificity for RIF-R detection was comparable for both S+ and S–, estimated to be 94.4% (95% CI: 81.9–98.5) and 91.7% (95% CI: 64.6–98.5), respectively.

Of the 149 C+ (S+ or S−) sputum samples included in the primary analysis, 118 had INH P-DST results available and had *M. tuberculosis* detected by both MDRmDx and Ultra assays, and 78 of these were INH-R and 40 were INH-S. The MDRmDx assay had a sensitivity of 76/77 (98.7%, 95% CI: 93.0–99.8) for INH-R detection with one INH indeterminate result and a specificity of 38/38 (100%, 95% CI: 90.8–100) with two INH indeterminate results (Fig. S5; Table 2).

Our initial analysis revealed three samples that were RIF-S by the P-DST reference standard but RIF-R by both the MDRmDx and Ultra assays (Fig. S4). All three samples were INH-R by the P-DST reference. Some mutations in the *M. tuberculosis rpoB* gene are well known to appear RIF-R when P-DST testing is performed on solid media but RIF-S when tested using liquid MGIT media. Since these so-called “disputed” mutations are also known to be associated with poor treatment outcomes (52), they are widely recognized as indicating true RIF-resistance (53). As per protocol, we performed Sanger DNA sequencing on the PCR amplicons generated in the MDRmDx and Ultra cartridges of the three RIF-R discordant samples. We also sequenced three additional PCR amplicons as controls, with concordant “RIF-R Not Detected” results by both assays, which matched with the RIF-S P-DST results. All three discordant amplicons were found to have *rpoB* mutations corresponding to the known “disputed” mutations L430P (Fig. S6A), D435Y (Fig. S6B), and H445L (Fig. S6C). In contrast, Sanger sequencing of the concordant RIF-S tests all showed WT *rpoB* gene sequences (Fig. S6D through F). Given these results, the “sequence-adjusted” RIF-R specificity for the MDRmDx assay was recalculated to be 45/45 (100%, 95% CI: 92.1–100) with one indeterminate result. For the Ultra assay, RIF-R detection specificity was recalculated as 43/43 (100%, 95% CI: 91.8–100) with three indeterminate results) (Table 2).

## DISCUSSION

The World Health Organization has recommended the Ultra assay for use in the detection of RIF-R TB; however, this assay can occasionally produce RIF indeterminate results when testing *M. tuberculosis* with mutations in codons 431–433 (such as Q432L and Q432P) (49), thereby hindering therapeutic decision-making. The Ultra assay also does not target the non-RRDR *rpoB* I491F mutation, which is strongly associated with RIF-R and a cause of local outbreaks of RIF-R TB (22, 54, 55). *M. tuberculosis* I491F mutants can be difficult to detect by conventional P-DST (21), and we are not aware of any other molecular assay that detects this mutation. The Ultra assay is not designed to detect INH-R; however, there is increasing evidence that INH mono-resistance is associated with poor TB treatment outcomes (56), and others have reported that INH-R usually precedes the emergence of RIF-R or MDR in clinical strains (24, 57). We developed the prototype MDRmDx assay to address these gaps in the Ultra assay.

The MDRmDx assay combines MTB detection with more comprehensive RIF and INH resistance detection than is available in the Ultra assay. The Abbott RealTime MTB RIF/INH (58, 59), BD Max MDR-TB (60, 61), Hain Lifescience FluoroType MTBDR (Hain) (62), and Roche cobas MTB-RIF/INH (63, 64) tests use a variety of DNA extraction methods and workflows to identify mutations in the *M. tuberculosis rpoB*, *katG*, and *inhA* gene promoter. However, these assays do not detect *rpoB* I491F or *fabG1* L203L mutations, which account for 0.5% to 30% of RIF-R (54) and 3%–5% of all INH-R TB depending on testing locale, respectively (17). With integrated sample preparation, the MDRmDx assay is also simpler to use, and it is comparatively faster with a time to result of approximately 90 min compared with turnaround times of 150 min or more for these other assays (60). With additional optimization, we expect that the MDRmDX assay time to result can be reduced to approximately 65 min for TB-negative samples and 75 min for TB-positive samples, similar to the time to results for the Ultra assay.

Our tests of frozen clinical sputum samples suggest that the specificity and sensitivity of the MDRmDx and Ultra assays are likely to be comparable when studied in a clinical setting. In our primary analysis, the MDRmDx assay failed to detect the presence of *M. tuberculosis* in three samples that were trace positive using the Ultra assay; however, this difference in sensitivity was modest and not statistically significant, and the analytical sensitivity of both assays for H37Rv mc^2^6230 and BCG strain detection was virtually identical. The MDRmDx and Ultra assays also had comparable sensitivity and specificity for detecting RIF-R in the sputum sample study. There were three pDST RIF-S samples which were identified as RIF-R by both MDRmDx and Ultra assays. Examination of the T_m_ values generated by both the assays revealed values consistent with mutations in codons 430, 435, and 445, which are known to produce borderline but clinically significant RIF-R by pDST on solid media (65). Our amplicon sequencing results verified the presence of these three mutations in the *rpoB* gene, strongly suggesting that the “sequence adjusted” specificities of 100% for both tests are more likely to be a more accurate reflection of the true values of each assay in our study. The MDRmDx assay showed a 98.7% (93.0–99.8) sensitivity and 100% (90.8–100) specificity for detecting INH resistance, which is comparable to that of the Xpert XDR assay, which has been shown to have a sensitivity of 98.3% (95% CI: 95.8–99.3) and specificity of 95% (95% CI: 73.1–99.7) (41).

The Ultra assay provides a semi-quantitative measure of the mycobacterial burden in samples where MTB is detected (High, Medium, Low, Very low, and Trace). These measures are not directly available in the MDRmDx assay except for “Trace.” However, the analytic studies we performed demonstrate that the earliest *rpoB* probe *C_T_* from the MDRmDx assay is comparable to the *C_T_* of the same probe in the Ultra assay, within the expected limits of variability. MDRmDx and Ultra testing of the split sputum samples in our clinical sample study also produced comparable *C_T_* values (Fig. S7). This finding provides additional confidence that *rpoB* probe *C_T_* values can be used as an interchangeable semi-quantitative measure between the two assays. Thus, even without the semi-quantitative result output, monitoring the earliest *rpoB* probe *C_T_* in the MDRmDx assay should provide end users with comparable information as the semi-quantitative result output in Ultra assay.

This study has some limitations. First, we noted that the MDRmDx assay did not perform as well as the Xpert XDR assay to detect INH heteroresistance caused by *katG* S315T mutants in tests of INTROL mixtures, while both assays performed equally well at detecting this mutation in mixtures of WT and *katG* mutant *M. tuberculosis*. The reason for these discordant results is not clear but may be due to the artificial nature of the INTROLs, which contain recombinant segments of WT (WT INTROL) or INH-R causing mutations (Mut1 INTROL) in the *katG*, *inhA*, and *fabG1* genes. Second, our study was performed with a limited number of prototype assay cartridge lots, and the performance of a fully optimized assay made in production lots may be different. Of note, the fully optimized and commercially produced Xpert Ultra and XDR assays had improved performance compared to their earlier prototypes. Third, our study of clinical sputum samples included many that had been stored for 5 years or more. It is possible that these long storage times adversely affected assay performance due to degradation of the *M. tuberculosis* bacteria that they contained. We also selected our samples to include a higher proportion of drug-resistant and smear-negative samples than might be expected during a prospective clinical trial. Thus, our clinical sample study may not completely reflect the overall performance of the MDRmDx assay in a clinical setting. We mitigated this potential limitation by ensuring that our primary analysis only included samples that had been tested by both the MDRmDx and Ultra assays, which allowed for a valid comparison between the two assays. The number of clinical sputum samples was relatively small but represented a wide geographical representation of disease endemic settings. Thus, even if our study cannot fully replicate a prospective clinical validation, it is likely that the clinical performance of the MDRmDx assay will be comparable to that of the Ultra assay in studies with larger sample sets.

Our results strongly suggest that a future and further optimized version of the MDRmDx assay is expected to have the potential to identify TB patients infected with RIF mono-resistant and INH mono-resistant *M. tuberculosis* as well as MDR *M. tuberculosis* strains at near patient settings, which will aid early administration of appropriate TB treatments. This is the first sample-to-answer test system that targets the non-RRDR I491F mutation in the *rpoB* gene, to the best of our knowledge. Advanced development and widespread implementation of this or similar tests should help prevent both underdiagnosis and misdiagnosis of drug-resistant *M. tuberculosis*, thereby ensuring appropriate treatment and limiting the development of additional drug resistance.

## AFTER EPUB

*[This article was published on 30 March 2026 with Samarpita Roybardhan and Ritu Anwesa missing from the byline. The byline was updated in the current version, posted on 13 April 2026.]*

## SUPPLEMENTAL MATERIAL

Figure S1 jcm.01100-25-s0001.tif Exclusivity of the MDRmDx assay against non-tuberculosis mycobacteria (NTM).

Figure S2 jcm.01100-25-s0002.tif Dynamic range studies.

Figure S3 jcm.01100-25-s0003.tif Participant flow diagram: Accuracy of the MDRmDx and Ultra assays for detection of MTB (primary analysis).

Figure S4 jcm.01100-25-s0004.tif Participant flow diagram: Accuracy of MDRmDx and Ultra assays for detection of rifampicin resistance (primary analysis).

Figure S5 jcm.01100-25-s0005.tif Participant flow diagram: Accuracy of MDRmDx assay for detection of isoniazid resistance (primary analysis).

Figure S6 jcm.01100-25-s0006.tif Discrepancy sequencing analysis.

Figure S7 jcm.01100-25-s0007.tif Comparing assay cycle threshold (CT) results.

Legends for Figures S1 to S7 jcm.01100-25-s0008.docx Figure S1: Exclusivity of the MDRmDx assay against non-tuberculosis mycobacteria (NTM). Figure S2: Dynamic range studies. Figure S3: Participant flow diagram: Accuracy of MDRmDx and Ultra assays for detection of MTB (primary analysis). Figure S4: Participant flow diagram: Accuracy of MDRmDx and Ultra assays for detection of rifampicin resistance (primary analysis). Figure S5: Participant flow diagram: Accuracy of MDRmDx assay for detection of isoniazid resistance (primary analysis). Figure S6: Discrepancy sequencing analysis. Figure S7: Comparing assay cycle-threshold (CT) results.

Tables S1 to S7 jcm.01100-25-s0009.pdf Table S1: Mutations that were detected by MDRmDx assay. Table S2: MDRmDx assay evaluation of INH-R strains. Table S3A: Inclusivity testing with a diverse set of MTB lineages. Table S3B: Inclusivity testing with MTBC strains. Table S4A: Results of the exclusivity testing with different Mycobacterial species. Table S4B: Results of the exclusivity testing with Gram-positive and Gram-negative bacteria. Table S4C: Results of the exclusivity testing with *Candida* species. Table S5: NTM interference testing with 3x LoD BCG. Table S6: Characteristics of participants who provided sputum specimens. Table S7: Sensitivity and unadjusted and sequence-adjusted specificity estimates for secondary analysis of the clinical sample study (including all results that were valid for the MDRmDx assay, Ultra assay, or both assays).

Supplemental file jcm.01100-25-s0010.pdf Retrospective study of clinical sputum samples: Secondary analysis.

ASM does not own the copyrights to Supplemental Material that may be linked to, or accessed through, an article. The authors have granted ASM a non-exclusive, world-wide license to publish the Supplemental Material files. Please contact the corresponding author directly for reuse.
