# Supplementary figures and images for: A cartridge-based assay for improved detection of multidrug-resistant Mycobacterium tuberculosis directly from sputum
Source: J Clin Microbiol. 2026 Mar 30;64(5):e01100-25. doi: 10.1128/jcm.01100-25 (PMC13170168; doi:10.1128/jcm.01100-25)

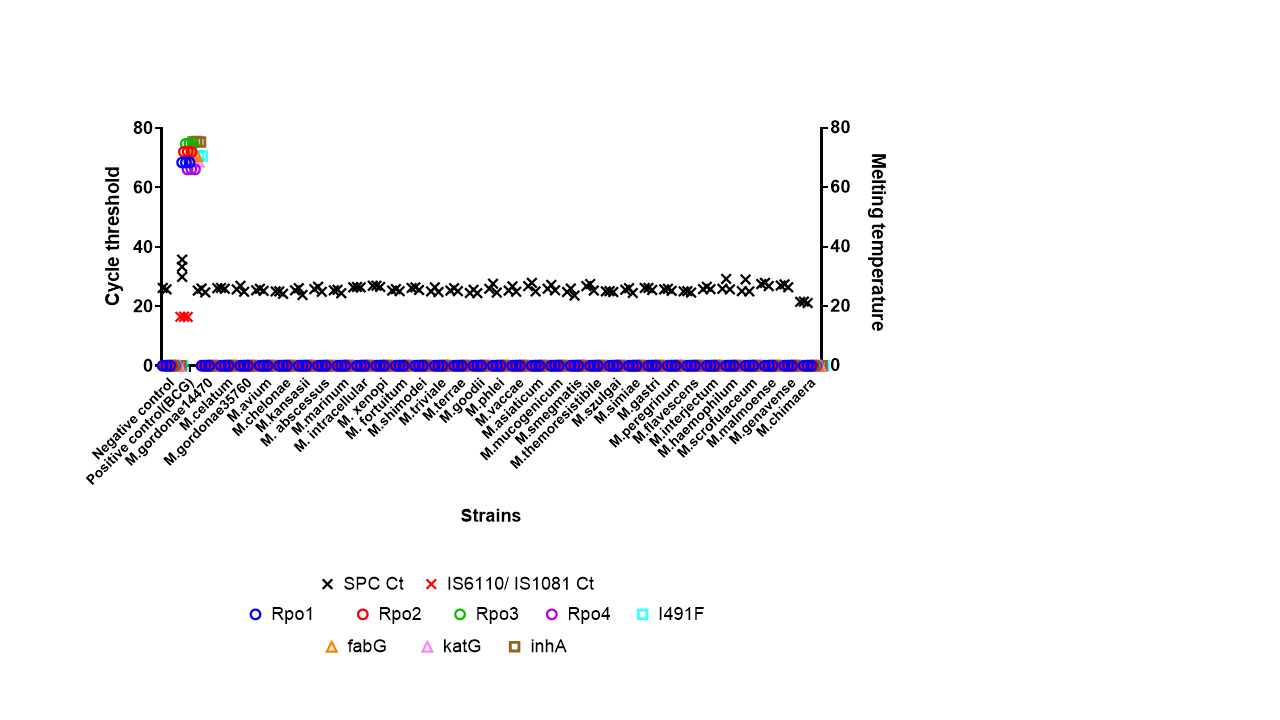

Supplement: Figure S1 — Exclusivity of the MDRmDx assay against non-tuberculosis mycobacteria (NTM). [file jcm.01100-25-s0001.tif]

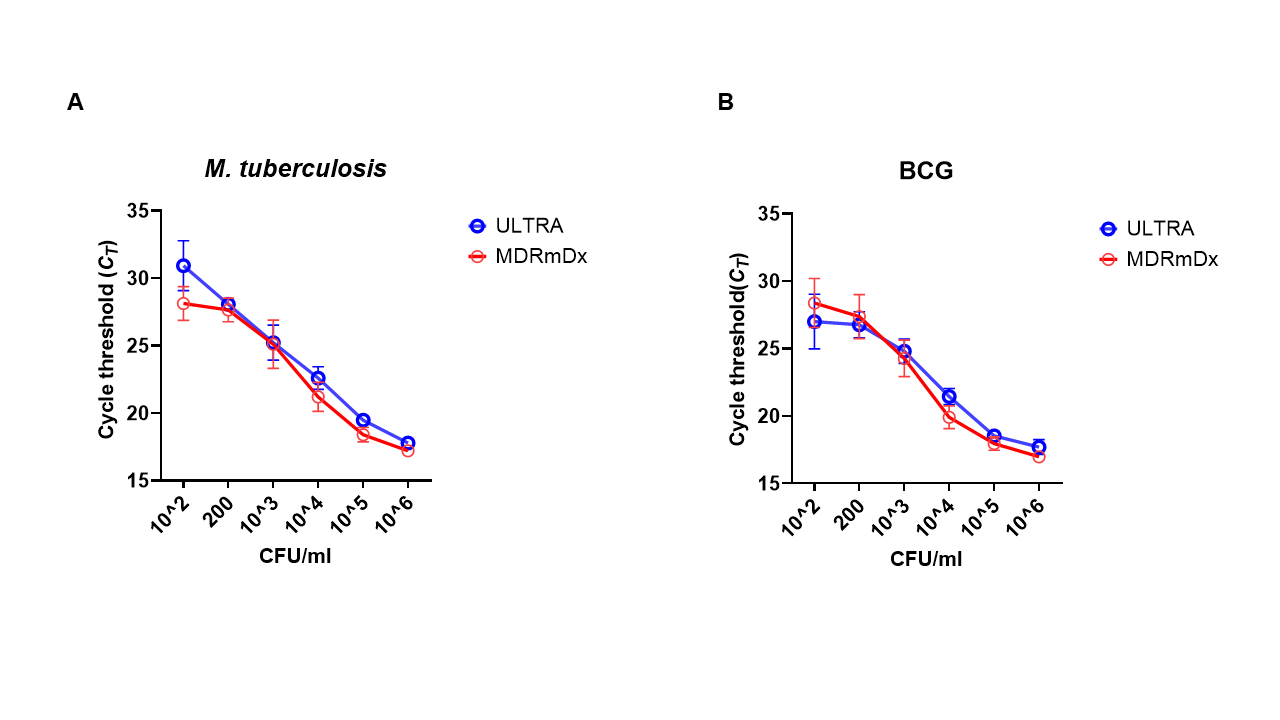

Supplement: Figure S2 — Dynamic range studies. [file jcm.01100-25-s0002.tif]

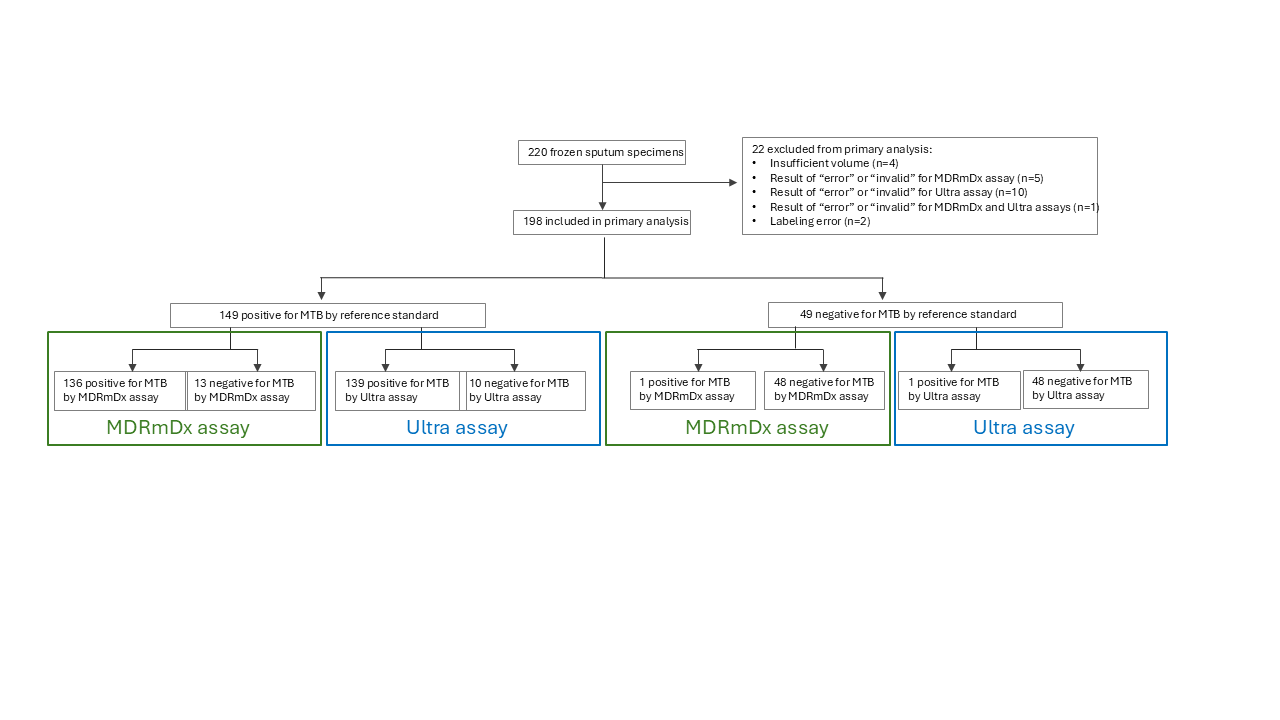

Supplement: Figure S3 — Participant flow diagram: Accuracy of the MDRmDx and Ultra assays for detection of MTB (primary analysis). [file jcm.01100-25-s0003.tif]

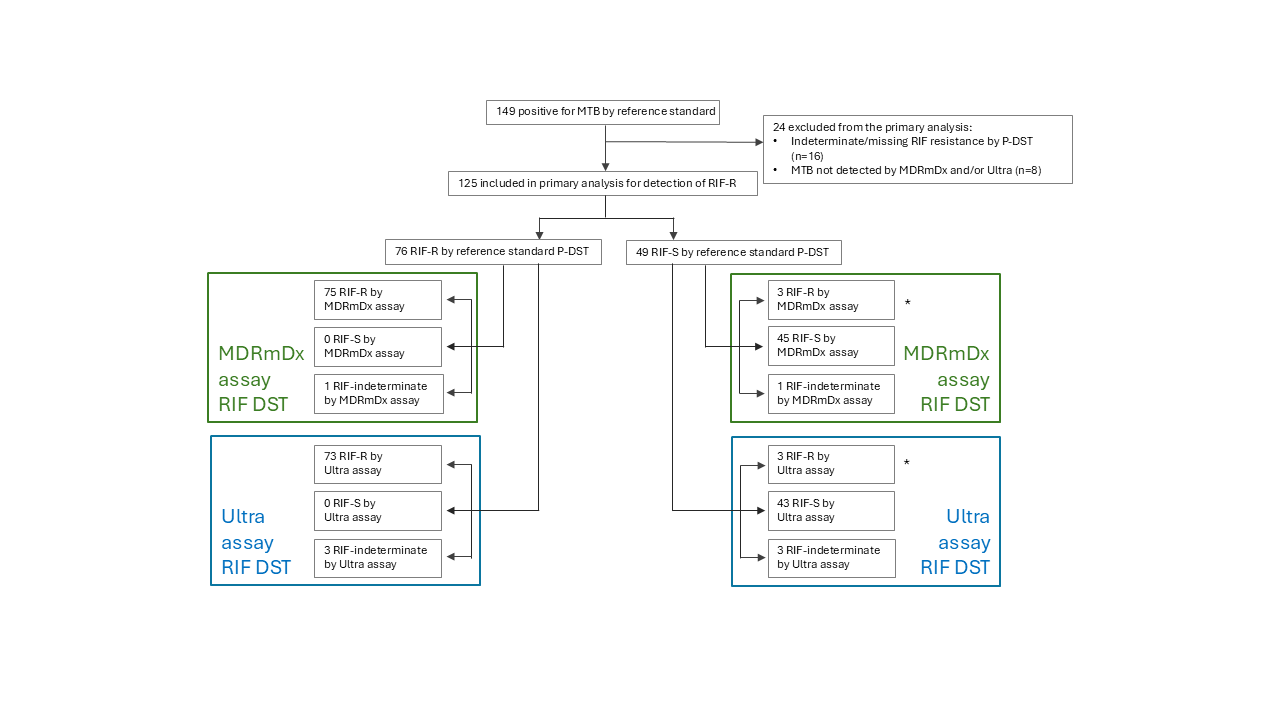

Supplement: Figure S4 — Participant flow diagram: Accuracy of MDRmDx and Ultra assays for detection of rifampicin resistance (primary analysis). [file jcm.01100-25-s0004.tif]

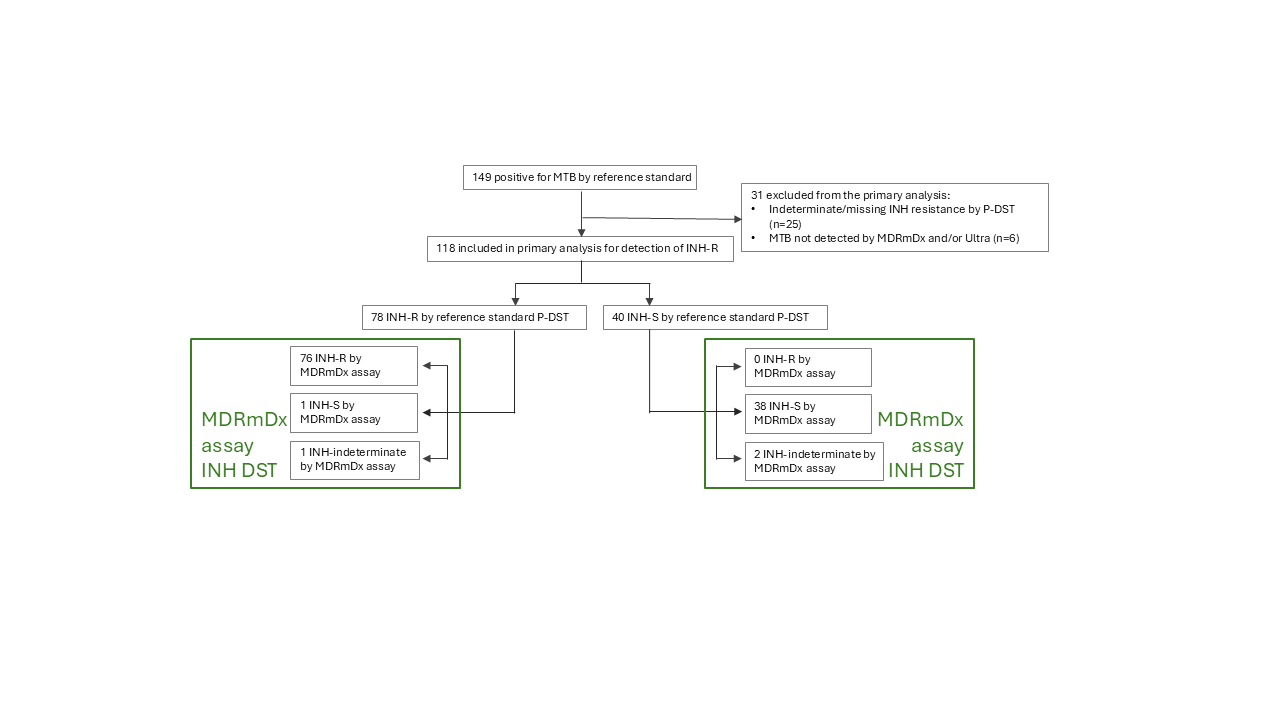

Supplement: Figure S5 — Participant flow diagram: Accuracy of MDRmDx assay for detection of isoniazid resistance (primary analysis). [file jcm.01100-25-s0005.tif]

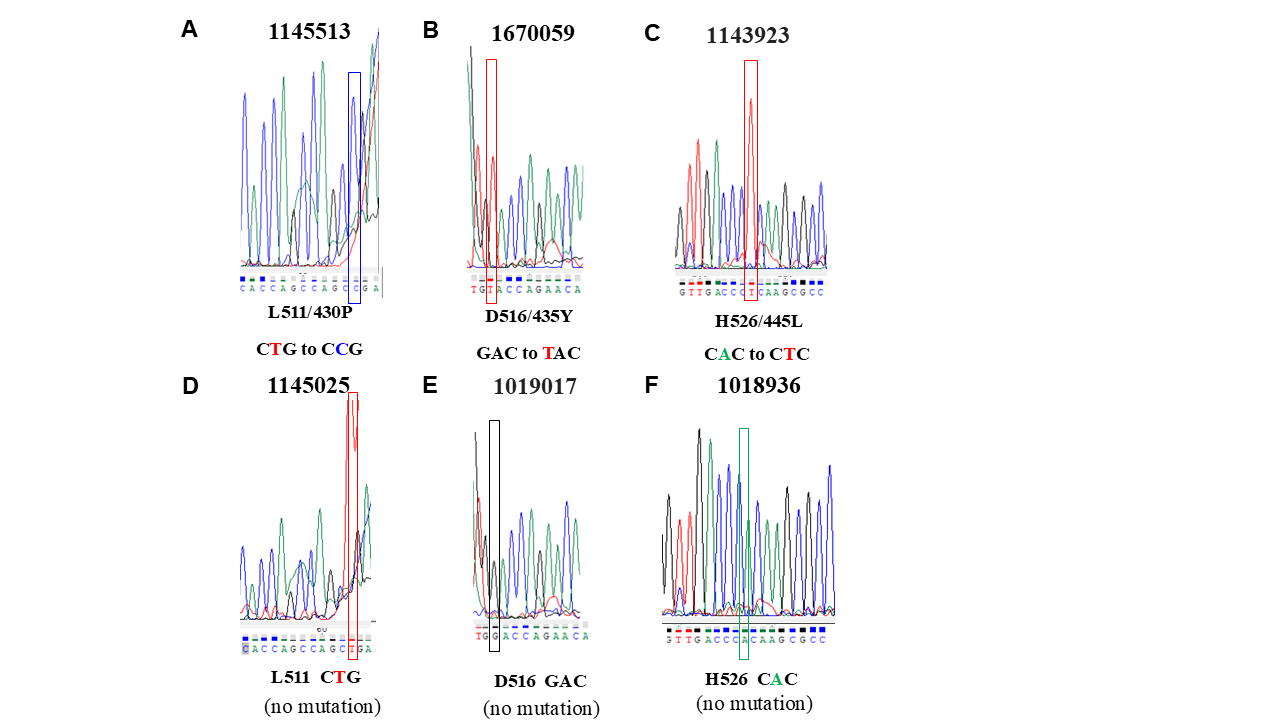

Supplement: Figure S6 — Discrepancy sequencing analysis. [file jcm.01100-25-s0006.tif]

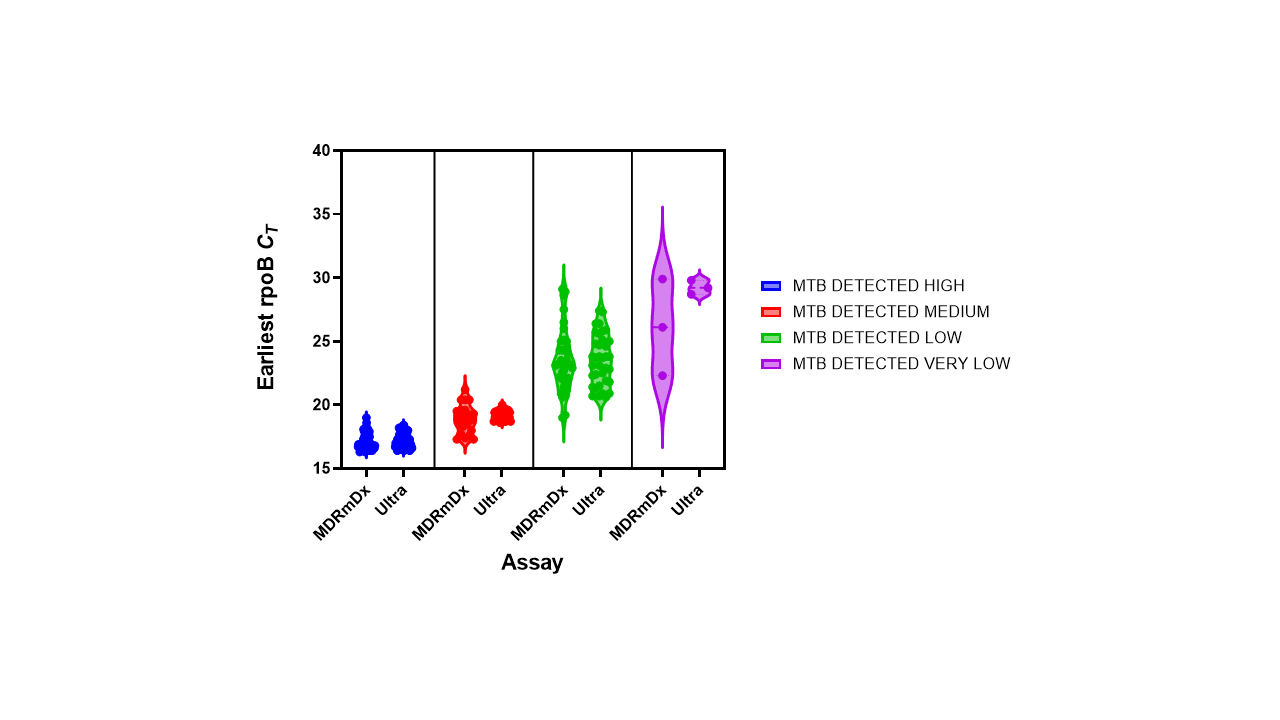

Supplement: Figure S7 — Comparing assay cycle threshold (CT) results. [file jcm.01100-25-s0007.tif]
