# Supplementary material for: A cartridge-based assay for improved detection of multidrug-resistant Mycobacterium tuberculosis directly from sputum
Source: J Clin Microbiol. 2026 Mar 30;64(5):e01100-25. doi: 10.1128/jcm.01100-25 (PMC13170168; doi:10.1128/jcm.01100-25)
Supplement: Legends for Figures S1 to S7 — Figure S1: Exclusivity of the MDRmDx assay against non-tuberculosis mycobacteria (NTM). Figure S2: Dynamic range studies. Figure S3: Participant flow diagram: Accuracy of MDRmDx and Ultra assays for detection of MTB (primary analysis). Figure S4: Participant flow diagram: Accuracy of MDRmDx and Ultra assays for detection of rifampicin resistance (primary analysis). Figure S5: Participant flow diagram: Accuracy of MDRmDx assay for detection of isoniazid resistance (primary analysis). Figure S6: Discrepancy sequencing analysis. Figure S7: Comparing assay cycle-threshold (CT) results. [file jcm.01100-25-s0008.docx]

**FIG S1**

**Exclusivity of the MDRmDx assay against non-tuberculosis mycobacteria (NTM).** Each sample was tested in three technical replicates with 10^6^ or 10^7^ CFU/mL of each indicated NTM. The cycle threshold (for the analytes SPC and *IS6110/IS1081*) and melting temperature values (for all the *rpoB* analytes as well as *inhA*, *fabG1* and *katG* analytes) are plotted for each of the NTM strains. BCG was used as positive control and buffer A as negative control.

**FIG S2**

**Dynamic range studies.** Comparative dynamic range studies of the MDRmDx (indicated by red line) and Ultra (indicated by blue line) assays are shown. Tenfold log dilutions of *M. tuberculosis* strain H37Rv mc^2^6230 (**A**) and BCG (**B**) spiked into *M. tuberculosis* negative sputum were tested in replicates of four for each dilution by both MDRmDx and Ultra. The earliest *rpoB* cycle threshold (*C_T_*) value for each log dilution were plotted against the concentration of the *M. tuberculosis* and BCG cells.

**FIG S3**

**Participant flow diagram: Accuracy of MDRmDx and Ultra assays for detection of MTB (primary analysis).**

Abbreviation: MTB, *Mycobacterium tuberculosis,* Ultra, Xpert MTF/RIF Ultra assay

**FIG S4. Participant flow diagram: Accuracy of MDRmDx and Ultra assays for detection of rifampicin resistance (primary analysis).**

Abbreviations: Ultra, Xpert MTB/RIF Ultra assay; MTB, *Mycobacterium tuberculosis;* P-DST, phenotypic drug susceptibility testing; RIF, rifampin; RIF-R, rifampicin resistant; RIF-S, rifampicin susceptible

* Samples that were confirmed to be RIF-R by targeted DNA sequencing

**FIG S5**

**Participant flow diagram: accuracy of MDRmDx assay for detection of isoniazid resistance (primary analysis).**

Abbreviations: MTB, *Mycobacterium tuberculosis;* P-DST, phenotypic drug susceptibility testing; INH, isoniazid; INH-R, isoniazid resistant; INH-S, isoniazid susceptible

**FIG S6**

**Discrepancy sequencing analysis.** Sanger DNA sequencing results for the three clinical sample study samples that were RIF-S by phenotypic DST samples but RIF-R by both MDRmDx and Ultra assays. The *rpoB* mutations that were identified by sequencing are indicated below each sequencing chromatogram. (**A**), (**B**) and (**C**) represent the mutations L511/430P, D516/435Y and H526/445L respectively. Sanger DNA sequencing results was done for the three clinical sample study samples that were RIF-S by phenotypic DST samples as well as by both MDRmDx and Ultra assays as controls. The *rpoB* sequence region of the control samples corresponding to each of the mutations in the discordants mentioned above are indicated below each sequencing chromatogram in (**D**), (**E**) and (**F**) respectively.

**FIG S7**

**Comparing assay cycle-threshold (*C_T_*) results.** Comparative analysis of the earliest *rpoB* *C_T_* for all of the clinical samples tested with both MDRmDx and Ultra assays are shown. Results are grouped by the Ultra semi-quantitative output measure as indicated by color used.
