# Supplementary material for: A cartridge-based assay for improved detection of multidrug-resistant Mycobacterium tuberculosis directly from sputum
Source: J Clin Microbiol. 2026 Mar 30;64(5):e01100-25. doi: 10.1128/jcm.01100-25 (PMC13170168; doi:10.1128/jcm.01100-25)
Supplement: Tables S1 to S7 — Table S1: Mutations that were detected by MDRmDx assay. Table S2: MDRmDx assay evaluation of INH-R strains. Table S3A: Inclusivity testing with a diverse set of MTB lineages. Table S3B: Inclusivity testing with MTBC strains. Table S4A: Results of the exclusivity testing with different Mycobacterial species. Table S4B: Results of the exclusivity testing with Gram-positive and Gram-negative bacteria. Table S4C: Results of the exclusivity testing with Candida species. Table S5: NTM interference testing with 3x LoD BCG. Table S6: Characteristics of participants who provided sputum specimens. Table S7: Sensitivity and unadjusted and sequence-adjusted specificity estimates for secondary analysis of the clinical sample study (including all results that were valid for the MDRmDx assay, Ultra assay, or both assays). [file jcm.01100-25-s0009.pdf]

Table S1: Mutations that were detected by MDRmDx assay.

Resistance-conferring variant	Tm (°C)(±SD)							
	rpo1	*rpo2*	*rpo3*	*rpo4*	*I491F*	*inhA*	*katG*	*fabG1*
None	68.7(0.25)	72.6(0.25)	75.3(0.2)	66.4(0.17)	70.9(0.2)	75.9(0.25)	69.2(0.25)	71(0.12)
L430P#	**64.9(0.06)**	**70.9(0.06)**	75.1(0.06)	66.4(0.06)	70.8(0.06)	**70.2(0)**	**62.6(0.06)**	70.9(0)
a1296g (Q432Q)	**67.4(0.06)**	74.4(0.15)	75.3(0.15)	66.3(0.06)	70.9(0.1)	75.8(0.15)	69.2(0.12)	71(0.1)
Q432L	**67.6(0.1)**	73(0.10)	75.6(0.06)	67.1(0)	71.2(0.12)	76.3(0.1)	**63.3(0.12)**	71.2(0.06)
Q432P	**67.3(0.06)**	72.6(0)	75.4(0.06)	66.7(0.1)	70.9(0.06)	75.9(0)	**62.9(0.06)**	71.1(0.06)
Q432K	**65.1(0.06)**	72.5(0.06)	75.4(0)	66.43(0.06)	70.9(0.06)	**70.4(0)**	69.3(0)	71(0)
D435V#	69.2(0.11)	**68.9(0.15)**	75.1(0.1)	66.5(0)	70.8(0.06)	**70.3(0.06)**	69.1(0.06)	71(0.06)
D435G#+I491F	70.3(0.2)	**71.3(0.15)**	75.4(0.15)	66.9(0.11)	**74.2(0.12)**	75.9(0.15)	**62.9(0.15)**	71.2(0.1)
D435L	69.3(0.12)	**69.1(0.11)**	75.2(0.1)	66.4(0.1)	70.7(*)	75.9(0.15)	**62.8(0.1)**	71(0.06)
D435Y#	69.3(0.17)	**68.5(0.11)**	75.3(0.15)	66.6(0.17)	70.9(0.06)	76.1(0.21)	**62.9(0.15)**	71.2(0.17)
S441L+ S450A	69.4(0.2)	**69.9(0.2)**	**72.5(0.15)**	67.2(0.06)	71(0.1)	76.1(0.2)	**63.1(0.2)**	71.2(0.15)
Q432L + G442E	**67.1(0.12)**	**70.7(0.2)**	**71.4(0.06)**	66.1(0.12)	70.9(0.1)	**70.4(0.15)**	69.2(0.12)	71.1(0.06)
H445R#	68.8(0.06)	72.9(0.06)	**73.7(0.06)**	68.5(0.2)	71.1(0)	75.9(0.06)	**63.5(0)**	71(0.06)
H445Y#	68.7(0)	72.6(0.10)	**71.9(0.06)**	66.7(0.06)	70.8(0.06)	75.8(0.06)	69.1(0.06)	70.9(0)
H445P	68.9(0.1)	72.9(0.06)	**73.8(0.06)**	68.9(0.06)	71.1(0)	75.9(0.06)	**62.9(0.06)**	70.9(0.06)
H445Stop+I491F	68.6(0.06)	72.4(0.10)	**71(0.06)**	66.7(0.06)	**73.9(0.06)**	**70.2(0.06)**	**62.6(0.1)**	70.9(0.06)
H445D#	68.6(0.11)	72.6(0.10)	**71.6(0.06)**	67.2(0.12)	70.9(0.1)	75.8(0.06)	**62.7(0.1)**	70.9(0.06)
H445S	68.7(0.06)	72.8(0.10)	**70.9(0.06)**	68.1(0.06)	70.9(0.06)	75.8(0.12)	69.2(0.06)	71.1(0)
H445C	68.8(0)	72.8(0.06)	**71.1(0)**	67.7(0.06)	71.1(0.06)	75.9(0.06)	**63.1(0.06)**	70.9(0.06)
H445L#	68.8(0.12)	72.7(0.15)	**71.9(0.06)**	66.7(0.12)	71(0.1)	75.8(0.12)	**62.8(0.1)**	70.9(0.06)
S431T+M434I + H445N	**61.5(0.06)**	**69.8(0.06)**	**72.3(0.06)**	66.9(0.06)	71(0.06)	76.2(0.06)	**63.2(0.06)**	71.3(0)
R448K	68.9(0.06)	72.4(0.06)	**74.2(0.06)**	**63.9(0)**	70.9(0.06)	**70.5(0.06)**	**62.8(0.1)**	71(0)
S450F#	68.7(0.12)	72.7(0.06)	**71.3(0.06)**	67.3(0)	70.9(0.1)	**70.3(0.06)**	**62.7(0.1)**	70.9(0.06)
S450W#	68.9(0.1)	73.3(0.15)	**73.1(0.2)**	**70.3(0.15)**	71(0.1)	75.9(0.15)	**62.9(0.12)**	71.2(0.1)
S450L#	68.8(0.12)	74.3(0.15)		**73.6(0.3)**	70.9(0.1)	75.9(0.17)	**62.8(0.15)**	71.1(0.12)
L449M + S450P	69.1(0.15)	73.2(0.2)	**69(0.1)**	**63.8(0.15)**	71.1(0.12)	**74.2(0.2)**	**63.1(0.15)**	71.2(0.13)
L452P#	68.8(0.35)	72.5(0.3)	75.3(0.3)	**60.8(0.2)**	70.9(0.2)	75.9(0.3)	**62.8(0.3)**	71.1(0.2)
P454P	68.7(0.06)	72.4(0.12)	75.2(0)	66.3(0.12)	70.8(0.06)	75.7(0.12)	69.1(0.06)	70.9(0.06)
I491F#	68.7(0)	72.5(0.06)	75.4(0.06)	66.6(0.06)	**73.9(0.06)**	75.8(0.06)	69.1(0.06)	70.9(0.06)

An asterisk (#) indicates mutations with a prevalence of at ≥ 0.5% as in the WHO 2023 catalogue. None = WT DNA tested.

* For D435L strain, the SD is not mentioned for the I49IF assay as only one of the 3 replicates gave I491F Tm while the other two were missed probably due to concentration of the sample being lower than the LoD for I491F detection.

Table S2: MDRmDx assay evaluation of INH-R resistant strains.

Strain name	pDST	Gene conferring resistance	Resistance conferring variant	MDRmDx result output	Tm (°C) (±SD)		
					inhA	***katG***	***fabG1***
BCG	NA	NA	None	MTB DETECTED, INH RESISTANCE NOT DETECTED	75.9(±0.25)	69.2(±0.25)	71.1(±0)
TDR-0073	INH-R	*katG*	S315N	MTB DETECTED, INH RESISTANCE DETECTED	75.9(±0.06)	**63.5 (±0)**	71.1(±0.06)
TDR-0021	INH-R	*katG*	S315T	MTB DETECTED, INH RESISTANCE DETECTED	76.1(±0.21)	**62.9(±0.15)**	71.2(±0.17)
OB079	INH-R	*katG*	S315R	MTB DETECTED, INH RESISTANCE DETECTED	75.9(±0.15)	**64.8(±0.1)**	70.9(±0.1)
OB160	INH-R	*inhA promoter, katG*	T(−8)A, S315T	MTB DETECTED, INH RESISTANCE DETECTED	**72.5(±0.1)**	**62.8(±0.06)**	70.9(±0)
TDR-0065	INH-R	*inhA promoter, katG*	T(−8)G, S315T	MTB DETECTED, INH RESISTANCE DETECTED	**72.4(±0.15)**	**62.7(±0.15)**	70.9(±0.1)
OB124	INH-R	*inhA promoter*	T(−8)C, S315T	MTB DETECTED, INH RESISTANCE DETECTED	**73.7(±0.06)**	69(±0.06)	70.9(±0.06)
TDR-0086	INH-R	*inhA promoter*	C(−15)T	MTB DETECTED, INH RESISTANCE DETECTED	**70.3(±0.06)**	69.1(±0.06)	71(±0.06)
TDR-0089	INH-R	*inhA promoter*	G(−9)A	MTB DETECTED, INH RESISTANCE DETECTED	**72.5(±0.1)**	69(±0.15)	70.8(±0.1)
OB202	INH-R	*fabG1*	L203L	MTB DETECTED, INH RESISTANCE DETECTED	75.8(±0.06)	69.1(±0.06)	**75.6(±0.06)**

Table S3A: Inclusivity testing with diverse set of MTB lineages.

Mycobacterium tuberculosis (BCCM#)	Lineage	No. of IS6110 copies	Results	MTB results	RIF susceptibility results	INH susceptibility results
BCCM_082	1	11	3/3 (100%)	MTB detected	RIF resistance not detected	INH resistance not detected
BCCM_087	2	16	3/3 (100%)	MTB detected	RIF resistance not detected	INH resistance not detected
BCCM_089	3	13	3/3 (100%)	MTB detected	RIF resistance not detected	INH resistance not detected
BCCM_093	4	11	3/3 (100%)	MTB detected	RIF resistance not detected	INH resistance not detected
BCCM_095	5	10	3/3 (100%)	MTB detected	RIF resistance not detected	INH resistance not detected
BCCM_100	6	5	3/3 (100%)	MTB detected	RIF resistance not detected	INH resistance not detected
BCCM_101	7	1	3/3 (100%)	MTB detected	RIF resistance not detected	INH resistance not detected

BCCM: Belgian Coordinated Collections of Microorganism

3x or above WT LoD were used

Table S3B: Inclusivity testing with MTBC strains.

***Mycobacterium tuberculosis* Complex (MTBC) strains**	**Results**	**MTB results**	**RIF susceptibility results**	**INH susceptibility results**
Mycobacterium bovis (27149)	3/3 (100%)	MTB detected	RIF resistance not detected	INH resistance not detected
Mycobacterium africanum(35711)	3/3 (100%)	MTB detected	RIF resistance not detected	INH resistance not detected
Mycobacterium canetti	3/3 (100%)	MTB detected	RIF resistance not detected	INH resistance not detected
Mycobacterium microti(19422)	3/3 (100%)	MTB detected	RIF resistance not detected	INH resistance not detected

3x or above WT LoD were used

Table S4A: Results of the exclusivity testing with different Mycobacterial species.

Sample	Strain ID	Results	MTB results	RIF susceptibility results	INH susceptibility results
Mycobacterium bovis (BCG)	-	12/12 (100%)	MTB detected	RIF resistance not detected	INH resistance not detected
Mycobacterium marinum	*ATCC # 0927*	0/3 (100%)	MTB Not detected		
Mycobacterium gordonae	*ATCC 14470*	0/3 (100%)	MTB Not detected		
Mycobacterium gordonae	*ATCC 35760*	0/3 (100%)	MTB Not detected		
Mycobacterium intracellulare	*ATCC # 35771*	0/3 (100%)	MTB Not detected		
Mycobacterium avium	*ATCC 15769*	0/3 (100%)	MTB Not detected		
Mycobacterium celatum	*ATCC# 51131*	0/3 (100%)	MTB Not detected		
Mycobacterium chelonae	*NJH*	0/3 (100%)	MTB Not detected		
Mycobacterium kansasii	*ATCC # 12478*	0/3 (100%)	MTB Not detected		
Mycobacterium xenopi	*ATCC # 700084*	0/3 (100%)	MTB Not detected		
Mycobacterium abscessus	*ATCC # 19977*	0/3 (100%)	MTB Not detected		
Mycobacterium haemophilum	*ATCC # 29548*	0/3 (100%)	MTB Not detected		
Mycobacterium asiaticum	*ATCC # 25276*	0/3 (100%)	MTB Not detected		
Mycobacterium flavescens	*ATCC # 23008*	0/3 (100%)	MTB Not detected		
Mycobacterium gastri	*ATCC # 15754*	0/3 (100%)	MTB Not detected		
Mycobacterium scrofulaceum	*ATCC # 19981*	0/3 (100%)	MTB Not detected		

Mycobacterium simiae	*ATCC # 25275*	0/3 (100%)	MTB Not detected
Mycobacterium szulgai	*ATCC # 23069*	0/3 (100%)	MTB Not detected
Mycobacterium thermoresistibile	*ATCC # 19527*	0/3 (100%)	MTB Not detected
Mycobacterium triviale	*ATCC # 23292*	0/3 (100%)	MTB Not detected
Mycobacterium vaccae	*NJH*	0/3 (100%)	MTB Not detected
Mycobacterium smegmatis	*IISc, Bangalore, India*	0/3 (100%)	MTB Not detected
Mycobacterium interjectum	*NJH*	0/3 (100%)	MTB Not detected
Mycobacterium peregrinum	*ATCC 700686*	0/3 (100%)	MTB Not detected
Mycobacterium mucogenicum	*NJH*	0/3 (100%)	MTB Not detected
Mycobacterium goodii	*NJH*	0/3 (100%)	MTB Not detected
Mycobacterium shimodei	*NJH*	0/3 (100%)	MTB Not detected
Mycobacterium phlei	*NJH*	0/3 (100%)	MTB Not detected
Mycobacterium terrae	*ATCC 15755*	0/3 (100%)	MTB Not detected
Mycobacterium genavense (**synthetic DNA**)	-	0/3 (100%)	MTB Not detected
Mycobacterium malmoense ***(synthetic DNA)***	-	0/3 (100%)	MTB Not detected
Mycobacterium chimaera	*BEI NR-49072*	0/3 (100%)	MTB Not detected
Negative control		0/12 (0%)	MTB Not detected

10e6 or 10e7 genomic copies or cells were used

Table S4B: Results of the exclusivity testing with Gram positive and Gram negative bacteria.

Sample	Strain ID	Results	MTB results
Klebsiella pneumoniae	*ATCC # 35657*	0/3 (100%)	MTB Not detected
Staphylococcus aureus	*ATCC # 25923*	0/3 (100%)	MTB Not detected
Streptococcus pyogenes	*ABC020063118, NR-48702* (BEI)	0/3 (100%)	MTB Not detected
Streptococcus viridans	*Ward's® #470179-178*	0/3 (100%)	MTB Not detected
Staphylococcus epidermis	*SK135, HM-118* (BEI)	0/3 (100%)	MTB Not detected
Nocardia asteroides (DNA)	*ATCC #19247*	0/3 (100%)	MTB Not detected
Escherichia coli CDC9707 (DNA)	NR-2651 bei, LOT:5107288	0/3 (100%)	MTB Not detected
Streptococcus agalactiae (DNA)	clinical DNA sample	0/3 (100%)	MTB Not detected
Streptococcus pneumoniae	*ATCC # 49619*	0/3 (100%)	MTB Not detected
Haemophilus influenzae	*ATCC # 10211*	0/3 (100%)	MTB Not detected
Negative control		0/6 (0%)	MTB Not detected

10e6 genomic copies or cells were used

Table S4C: Results of the exclusivity testing with Candida species.

Sample	Strain ID	Results	MTB results
Candida albicans	*ATCC # 470547*	0/3 (100%)	MTB Not detected
Candida krusei	*ATCC # 6258*	0/3 (100%)	MTB Not detected
Candida auris	*AR 0388*	0/3 (100%)	MTB Not detected
Candida glabrata	*ATCC # 15126*	0/3 (100%)	MTB Not detected
Candida parapsilosis	*ATCC # 22019*	0/3 (100%)	MTB Not detected
Candida tropicalis	*ATCC # 66029*	0/1(100%)*	MTB Not detected
Negative control		0/3 (0%)	MTB Not detected

"*" 2/3 tests were invalid for *Candida tropicalis.*

Table S5: NTM interference testing with 3× LoD BCG.

Sample	Results	MTB results	RIF susceptibility results	INH susceptibility results
BCG	3/3 (100%)	MTB detected	RIF resistance not detected	INH resistance not detected
Mycobacterium marinum + BCG	3/3 (100%)	MTB detected	RIF resistance not detected	INH resistance not detected
**Mycobacterium gordonae* ATCC 14470 + BCG	2/2 (100%)	MTB detected	RIF resistance not detected	INH resistance not detected
Mycobacterium gordonae ATCC 35760 + BCG	3/3 (100%)	MTB detected	RIF resistance not detected	INH resistance not detected
Mycobacterium intracellulare + BCG	3/3 (100%)	MTB detected	RIF resistance not detected	INH resistance not detected
Mycobacterium avium + BCG	3/3 (100%)	MTB detected	RIF resistance not detected	INH resistance not detected
Mycobacterium chimaera+ BCG	3/3 (100%)	MTB detected	RIF resistance not detected	INH resistance not detected
Mycobacterium kansasii + BCG	3/3 (100%)	MTB detected	RIF resistance not detected	INH resistance not detected
Mycobacterium abscessus + BCG	3/3 (100%)	MTB detected	RIF resistance not detected	INH resistance not detected
Negative control	0/3 (0%)	MTB Not detected		

* ERROR code 2005: Motion of syringe drive was not detected

Table S6. Characteristics of participants who provided sputum specimens.*

	Primary Analysis Group N=198	All Participants N=220
**Sex**		
Male	128 (65%)	143 (65%)
Female	66 (33%)	73 (33%)
Missing	4 (2%)	4 (2%)
**Enrollment Country**		
Cambodia	1 (<1%)	1 (<1%)
Georgia	23 (12%)	26 (12%)
Moldova	32 (16%)	35 (16%)
Peru	63 (32%)	69 (31%)
South Africa	33 (17%)	37 (17%)
Vietnam	46 (23%)	52 (24%)
**Enrollment Year**		
2013	3 (2%)	3 (1%)
2014	13 (7%)	17 (8%)
2015	29 (15%)	31 (14%)
2016	48 (24%)	54 (25%)
2017	68 (34%)	72 (33%)
2018	9 (5%)	12 (5%)
2019	12 (6%)	13 (6%)
2020	4 (2%)	4 (2%)
2021	11 (6%)	13 (6%)
Missing	1 (1%)	1 (<1%)
**HIV status**		
Positive	13 (7%)	13 (6%)
Negative	99 (50%)	111 (50%)
Missing	86 (43%)	96 (44%)
**MTB Status based on reference standard**		
Positive for MTB	149 (75%)	165 (75%)
Negative for MTB	49 (25%)	55 (25%)
**Rifampicin resistance status based on reference standard**	80 (40%)	89 (41%)
Resistant	53 (27%)	58 (26%)
Susceptible	65 (33%)	73 (33%)
Missing		

**Isoniazid resistance status based on reference standard**		
Resistant	82 (41%)	93 (42%)
Susceptible	42 (21%)	44 (20%)
Missing	74 (37%)	83 (38%)

*Sum of column percentages to not always add to 100% due to rounding.

Table S7: Sensitivity and unadjusted and sequence-adjusted specificity estimates for secondary analysis of the clinical sample study (including all results that were valid for either the MDRmDx, Ultra assays, or both assays).

	**MDRmDx / Reference Standard Results**				**Sensitivity**		**Specificity**	
	+/+	+/−	−/+	−/−	**%**	**95% CI**	**%**	**95% CI**
MTB detection	141	1	17	54	89.2	83.4 – 93.2	98.2	90.4 – 99.7
Among Smear+	102	0	2	0	98.1	93.3 – 99.5	N/A	N/A
Among Smear−	39	1	15	54	72.2	59.1 – 82.4	N/A	N/A
RIF	79	3	0	46	100	95.4 – 100	93.9	83.5 – 97.9
Among Smear+	56	2	0	35	100	93.6 – 100	94.6	82.3 – 98.5
Among Smear−	23	1	0	11	100	85.7 – 100	91.7	64.6 – 98.5
RIF (sequence adjusted)	82	0	0	46	N/A	N/A	100	92.3 – 100
INH	80	0	1	39	98.8	93.3 – 99.8	100	91.0 – 100
Among Smear+	58	0	1	29	98.3	91.0 – 99.7	100	88.3 – 100
Among Smear−	22	0	0	10	100	85.1 – 100	100	72.2 – 100

	**Ultra / Reference Standard Results**				**Sensitivity**		**Specificity**	
	+/+	+/−	−/+	−/−	**%**	**95% CI**	**%**	**95% CI**
MTB detection	145	1	10	48	93.5	88.5 – 96.5	98.0	89.3 – 99.6
Among Smear+	102	0	2	0	98.1	93.3 – 99.5	N/A	N/A
Among Smear−	43	1	8	48	84.3	72.0 – 91.8	N/A	N/A
RIF	77	3	0	44	100	95.2 – 100	93.6	82.8 – 97.8
Among Smear+	56	2	0	35	100	93.6 – 100	94.6	82.3 – 98.5
Among Smear−	21	1	0	9	100	84.5 – 100	90.0	59.6 – 98.2
RIF (sequence adjusted)	80	0	0	44	100	N/A	100	92.0 – 100

For MTB detection: "+" denotes MTB detected and "−" denotes MTB not detected.

For drug resistance: "+" denotes resistance detected and "−" denotes no resistance detected.

+/+: positive by both assay and reference standard.

+/−: positive by assay and negative by reference standard.

−/+: negative by assay and positive by reference standard.

−/−: negative by both assay and reference standard.

95% CIs are calculated using Wilson score method.

Abbreviations: MTB, *Mycobacterium tuberculosis*; N/A, not applicable.
