# Supplementary material for: A cartridge-based assay for improved detection of multidrug-resistant Mycobacterium tuberculosis directly from sputum
Source: J Clin Microbiol. 2026 Mar 30;64(5):e01100-25. doi: 10.1128/jcm.01100-25 (PMC13170168; doi:10.1128/jcm.01100-25)
Supplement: Supplemental file — Retrospective study of clinical sputum samples: Secondary analysis. [file jcm.01100-25-s0010.pdf]

Retrospective study of clinical sputum samples - Secondary Analysis

We also performed a secondary analysis to analyze the performance of each assay separately, including all the sputum samples that were tested and produced valid results with the specific assay under consideration. MGIT and P-DST reference standards, and additionally Sanger sequencing as reference for discordant results, were used in the same way as in the primary analysis. For the MDRmDx assay, 7 of the 220 (3.2%) study samples were excluded from the secondary analysis. Six excluded samples resulted in an "error" when tested with the MDRmDx assay. One sample was not tested with the MDRmDx assay due to labeling errors. One sample was tested twice by the MDRmDx assay also due to labeling errors; both tests of the same sample produced identical results and only one was included in the secondary analysis. Also included in the secondary analysis were four samples that were tested only with MDRmDx assay due to low volumes (less than 1500 µl) as per protocol. The overall diagnostic sensitivity of the MDRmDx assay for MTB detection was 141/158 (89.2%, 95% CI, 83.4 – 93.2) and the specificity was 54/55 (98.2%, 95% CI, 90.4 – 99.7). For C+/S+ sputum, the sensitivity of MTB detection was 102/104 (98.1%, 95% CI, 93.3 – 99.5), while for C+/S− sputum, the sensitivity for MTB detection was 39/54 (72.2%, 95% CI, 59.1 – 82.4) (Table S7).

Of the 213 samples included in the secondary analysis of the MDRmDx assay, 158/213 (74.2%) were C+ (S+ or S−), of which 140/158 (88.6%) had RIF P-DST results, and of these 84/140 (60%) were RIF-R by P-DST. The sensitivity for MDRmDx assay for RIF-R detection was 79/79 (100%, 95% CI, 95.4 – 100) with one RIF indeterminate result, and four with an "MTB Not Detected" result. The specificity for RIF-R detection was 46/49 (93.9.%, 95% CI, 83.5 – 97.9) with one RIF indeterminate, and six with an "MTB Not Detected" result (Table S7). The "sequence-adjusted" RIF-R sensitivity was 82/82 (100%, 95% CI, 95.5 – 100).

Of the 158 C+ (S+ or S−) sputum samples included in the secondary analysis, 120 had INH drug susceptibility results available and 87 (67%) of these were INH-R. The MDRmDx assay had sensitivity of 80/81 (98.8%, 95% CI, 93.3 – 99.8) for INH-R detection with one INH indeterminate results, and five with an "MTB Not Detected" result, and a specificity of 39/39 (100%, 95% CI, 91.0 – 100) with two INH indeterminate samples and two with an "MTB Not Detected" result (Table S7).

For the Ultra assay, 16 of the 220 (7.3%) study samples were excluded from the secondary analysis. Four samples were not tested with the Ultra assay due to their low volume, which only permitted testing with the MDRmDx assay. Ten excluded samples produced an "Error" result. An additional excluded sample produced an "Invalid" result. One sample was not tested with the Ultra assay due to labeling errors. One sample was tested twice by the Ultra assay also due to labeling error, both produced identical results and only one was included in the secondary analysis. The overall diagnostic sensitivity of the Ultra assay for MTB detection was 145/155 (93.5%, 95% CI, 88.5 – 96.5). Specificity for MTB detection was 48/49 (98.0 %, 95% CI, 89.3 – 99.6). For C+/S+ sputum, the sensitivity of Ultra MTB detection was 102/104 (98.1%, 95% CI, 93.3 – 99.5). For C+/S− sputum, the sensitivity of Ultra for MTB detection was 43/51 (84.3%, 95% CI, 72.0 – 91.8) (Table S7).

Of the 204 samples included in the secondary analysis of the Ultra assay, 155/204 (75.9%) were C+ (S+ or S−), of which 139/155 (89.7%) had RIF P-DST results, and 84/139 (60.4%) of these were RIF-R. The sensitivity for RIF-R detection was 77/77, (100%, 95% CI, 95.2 – 100) with four RIF indeterminate samples and three samples with an "MTB Not Detected" result. The specificity for RIF-R detection was 44/47 (93.6 %, 95% CI, 82.8 – 97.8) with six RIF indeterminate samples and two samples with an "MTB Not Detected" result (Table S7). Using DNA sequencing as a

reference, a “sequence-adjusted” RIF-R detection specificity was 47/47 (100%, 95% CI, 92.4 – 100) (Table S7). The assay “nondeterminate rate” (Errors plus Invalid results) for the Ultra assay was 5.1% (11/215) whereas for the MDRmDx assay it was 2.7% (6/219).
